# SPP1 as a biomarker for idiopathic membranous nephropathy progression and its regulatory role in inflammation and fibrosis

**DOI:** 10.3389/fimmu.2025.1671891

**Published:** 2025-09-26

**Authors:** Shuting Pang, Rongbin Zhou, Zige Liu, Boji Xie, Fugang Liu, Bingmei Feng, Xuesong Chen, Liangping Ruan, Hong Chen, Yuli Xie, Qiuyan Tan, Binran Zhao, Shanshan Li, Chao Xue, Rirong Yang, Wei Li

**Affiliations:** ^1^ Department of Nephrology, The Second Affiliated Hospital of Guangxi Medical University, Nanning, Guangxi Zhuang, China; ^2^ Center for Genomic and Personalized Medicine, Guangxi key Laboratory for Genomic and Personalized Medicine, Guangxi Collaborative Innovation Center for Genomic and Personalized Medicine, University Engineering Research Center of Digital Medicine and Healthcare, Guangxi Medical University, Nanning, Guangxi Zhuang, China; ^3^ Institute of Urology and Nephrology, The First Affiliated Hospital of Guangxi Medical University, Guangxi Medical University, Nanning, Guangxi Zhuang, China; ^4^ Department of Immunology, School of Basic Medical Sciences, Guangxi Medical University, Nanning, Guangxi Zhuang, China

**Keywords:** idiopathic membranous nephropathy, proximal tubular cells, SPP1, fibrosis, inflammation

## Abstract

**Objective:**

Idiopathic membranous nephropathy (IMN) is a leading cause of nephrotic syndrome in middle-aged and elderly populations. Early intervention can delay disease progression and improve patient outcomes. This study aims to identify urinary biomarkers for IMN and investigate their association with disease progression, offering new insights for precise diagnosis and treatment.

**Methods:**

This study began with RNA sequencing of three urine sample types (first-void morning urine, second-void morning urine, and random urine), combined with single-cell RNA sequencing of renal tissues. Bioinformatics analyses—including differential gene expression screening, machine learning, and molecular function annotation—were employed to identify potential IMN biomarkers. Furthermore, we established both a siRNA-mediated gene silencing model and a lentivirus transfection-mediated gene overexpression model in HK-2 cells. Subsequently, we investigated the functional mechanisms of the candidate biomarkers through qRT-PCR, Western blot, immunohistochemistry, and immunofluorescence assays.

**Results:**

SPP1 was identified as a promising biomarker for IMN, demonstrating a critical role in promoting fibrosis and inflammatory responses associated with the disease. These findings suggest its potential as a novel therapeutic target for IMN intervention.

## 1 Introduction

Idiopathic Membranous Nephropathy (IMN) accounts for approximately 30% of adult nephrotic syndrome cases (1), with nearly 40% of patients progressing to end-stage renal disease (2, 3). Early detection and timely intervention can significantly improve outcomes while reducing the socioeconomic burden. Pathologically, IMN is characterized by immune complex deposition, complement-mediated proteinuria, and progressive renal impairment (4). Current clinical staging relies on histopathological features including the degree of glomerular basement membrane thickening, presence of spike formations, and immunoglobulin deposition patterns (5). These pathological classifications facilitate disease progression monitoring and prognosis prediction. The disease pathogenesis involves inflammatory cell infiltration and subsequent release of vasoactive mediators, leading to vascular hyperpermeability, leukocyte recruitment, and other inflammatory injuries (6). These processes represent critical pathological hallmarks that drive IMN progression to chronic kidney disease. Ultimately, renal fibrosis progression and sustained immune activation form the core pathological mechanisms in IMN development, suggesting that modulation of inflammatory responses may serve as a promising therapeutic strategy.

Genetic alterations are closely associated with disease pathogenesis. Accumulating evidence indicates that differentially expressed genes may serve as both reliable disease biomarkers and promising therapeutic targets (7). In recent years, RNA sequencing (RNA-seq), particularly single-cell RNA sequencing (scRNA-seq), has revolutionized our understanding of cellular heterogeneity and intercellular communication networks owing to its unparalleled capacity for comprehensive and precise gene expression profiling (8). This cutting-edge technology has substantially advanced research in renal pathophysiology, facilitating the identification of diagnostic biomarkers and discovery of novel therapeutic targets.

Urine contains various exfoliated renal cells with significant research value. Studies have demonstrated that urine from both healthy and diseased kidneys contains sufficient exfoliated proximal tubular cells (PTCs) that can reflect renal functional changes (9). Different urine sampling methods offer distinct advantages: first-void morning urine is the most clinically practical due to its convenience (10), while second-void morning urine-collected after initial bladder emptying-shows increased proportions of exfoliated renal cells with reduced contamination from urethral epithelial cells, thereby providing more accurate pathological information (11). Although random urine sampling offers convenience and temporal flexibility, its clinical application remains limited due to potential variability. To address these limitations, our study integrated RNA-seq data from all three urine sample types to identify consistently and highly expressed genes across different sampling conditions, aiming to discover more reliable urinary biomarkers. Notably, secreted phosphoprotein 1 (SPP1) emerged as a particularly promising candidate owing to its exceptional diagnostic performance.

SPP1, also known as Osteopontin, is encoded on chromosome 4 and expressed in bone tissue, renal tissue, and other tissues (12). Early studies considered SPP1 a calcium-binding protein involved in bone formation, playing a critical role in regulating bone mineralization (13). With in-depth research on SPP1, it has been found to participate in numerous biological processes such as inflammation and fibrosis, and to have the potential as a biomarker (14). In the brain, SPP1 can activate microglia to facilitate synaptic phagocytosis (15); in lung tissue, SPP1 promotes M2 macrophage polarization through the Jak2/Stat3 signaling pathway, accelerating the progression of idiopathic pulmonary fibrosis (16); in renal tissue, SPP1 acts as a “driver” of renal fibrosis and inflammatory progression (17, 18). Given these pleiotropic effects, elucidating SPP1’s mechanistic roles is essential for developing therapeutic strategies against kidney disease progression.

In this study, through RNA sequencing of urinary exfoliated cells from three sampling methods, we identified SPP1 as an exceptional biomarker for disease progression. Its expression levels showed a significant positive correlation with pathological severity in IMN patients. Histological and *in vitro* cellular experiments further demonstrated that SPP1 regulates the expression of inflammatory and fibrotic factors. These findings suggest that SPP1 not only serves as a potential diagnostic biomarker for IMN, but may also represent a novel therapeutic target for disease intervention.

## 2 Research methods and materials

### 2.1 Collection of samples and clinical information

PLA2R (M-type phospholipase A2 receptor) is a key autoantigen in the pathogenesis of IMN, and anti-PLA2R antibodies can be detected in the serum of approximately 70%-80% of IMN patients (19). Additionally, PLA2R serves as a specific diagnostic biomarker for IMN, which helps distinguish idiopathic membranous nephropathy from secondary membranous nephropathy (20).

In this study, the diagnostic criteria for membranous nephropathy were based on renal biopsy pathological results and the detection status of anti-PLA2R antibodies. Only patients who underwent renal biopsy for the first time and were pathologically diagnosed with IMN were included. All urine samples were collected before the patients underwent renal biopsy to avoid interference of the biopsy procedure on sample quality.

A total of 17 PLA2R antibody-positive IMN patients were enrolled in the study, with 47 urine samples collected cumulatively; meanwhile, 17 healthy volunteers were included as controls, with 51 urine samples collected cumulatively, which is consistent with the sequencing samples used in our previous study (DOI: 10.3389/fmed.2025.1574852).

The exclusion criteria were set as follows (1): Patients diagnosed with other types of kidney diseases (excluding IMN); (2) Patients with anuria or those requiring long-term dialysis (hemodialysis/peritoneal dialysis) treatment; (3) Patients with severe systemic diseases or major organ dysfunction, including but not limited to: 1. Severe cardiovascular diseases (e.g., heart failure with New York Heart Association [NYHA] Class III-IV, new-onset myocardial infarction within the past 6 months); 2. Severe liver diseases (e.g., liver cirrhosis with Child-Pugh Class B or higher, clinically confirmed active hepatitis); 3. Severe respiratory diseases (e.g., acute exacerbation of chronic obstructive pulmonary disease, respiratory failure requiring mechanical ventilation support); (4) Individuals with poor medical compliance who were unable to cooperate with sample collection and provide relevant information; (5) Individuals who refused to sign the informed consent form due to personal reasons.

The study protocol received ethical approval from the Second Affiliated Hospital of Guangxi Medical University (Approval No. 2023KY-0715) and was conducted in compliance with the Declaration of Helsinki, with written informed consent obtained from all participants or guardians.

For sample collection, patients provided first-void morning urine immediately upon waking, followed by second-void morning urine within 2 hours, and random urine samples between 12:00 and 20:00. All samples were immediately processed through centrifugation at 490×g for 10 minutes at 4 °C, with supernatants stored in 1.5 mL EP tubes (Axygen, USA) and cell pellets washed twice with chilled DPBS (Wisent, Canada) using centrifugation at 2,000 rpm for 5 minutes at 4 °C before final storage at -80 °C freezer. Clinical data were obtained from medical records and health examination reports at the Second Affiliated Hospital of Guangxi Medical University, with detailed parameters provided in **Supplementary Table 1**.

### 2.2 RNA sequencing of urinary exfoliated cells and primary analysis of raw sequencing data

Urinary cell pellets were retrieved from the -80 °C freezer, and bulk RNA sequencing was performed using the AccuraCode^®^ HTP One-Step RNA Sequencing Kit (Singleron Biotechnologies Co., Ltd., China). The specific steps were as follows: cell lysis and mRNA capture, ONE Step amplification to obtain cDNA, cDNA product purification, quality inspection of purified cDNA products, cDNA product fragmentation reaction, adapter ligation to fragmented products, purification of adapter-ligated products, PCR enrichment of purified products, transcriptome library sorting, transcriptome library quality inspection, and library sequencing.

After library preparation, stringent quality control (QC) was conducted, which included assessments of total library yield (>30 ng), fragment size distribution (main peak: 300–600 bp), large fragment contamination (fragments of 900–5000 bp accounting for <20%), and small fragment residues (fragments of <300 bp accounting for <20%). Qualified libraries were subjected to paired-end sequencing on the Illumina NovaSeq platform.

Raw reads were processed with Celescope (v2.0.7) using default parameters to generate gene expression profiles. Briefly, barcodes and unique molecular identifiers (UMIs) were extracted from R1 reads and subjected to error correction. Adapter sequences and poly-A tails were trimmed from R2 reads using cutadapt (v3.7). The trimmed R2 reads were then aligned to the Homo sapiens GRCh38.99 reference genome using STAR (v2.7.11a). Uniquely mapped reads were assigned to genes using featureCounts (v2.0.1). Finally, successfully assigned reads sharing identical barcodes, UMIs, and gene annotations were aggregated to generate the gene expression matrix for downstream analysis.

### 2.3 Integration of RNA-seq datasets and differential gene expression analysis

The bioinformatics analysis of RNA-seq data was performed using R (version 4.3.1; https://www.r-project.org/). Batch effects between samples were corrected using the sva package. Differential expression analysis between IMN patients and healthy volunteers across three urine sample types was conducted using edgeR with thresholds of |logFC| > 1 and *p < 0.05*. We then performed cross-analysis of differentially expressed genes (DEGs) from the three urine sample types, focusing on consistently dysregulated genes across all samples. The integrated DEGs were visualized using the ggplot2 package for volcano plots and the VennDiagram package for Venn diagrams.

### 2.4 Functional enrichment analysis of DEGs and protein-protein interaction network construction

Functional enrichment analysis was performed using the clusterProfiler package, which conducted Gene Ontology (GO) and Kyoto Encyclopedia of Genes and Genomes (KEGG) pathway enrichment analyses on the DEGs. The results were visualized using the ggplot2 package, while functional characterization of DEGs was further analyzed through gene set enrichment analysis (GSEA) implemented in the GSVA package. Protein-protein interaction (PPI) networks were constructed using the STRING database (https://string-db.org/). The commonly upregulated DEGs across all three urine sample types were subsequently visualized using Cytoscape software (version 3.8.2).

### 2.5 Machine learning-based identification of key biomarkers and evaluation of diagnostic performance

Three machine learning approaches - Random Forest, LASSO (Least Absolute Shrinkage and Selection Operator), and Support Vector Machine (SVM) - were employed to screen the consistently upregulated DEGs across all three urine sample types. The identified key biomarkers were subsequently used to construct a nomogram with the rms package, utilizing the “nomogram”, “validate”, and “calibrate” functions. To evaluate the diagnostic performance, receiver operating characteristic (ROC) curves were generated with the plotROC package, and the area under the curve (AUC) was calculated to assess the predictive power and validate the efficacy of these key biomarkers.

### 2.6 Functional exploration, regulatory mechanisms, and therapeutic targets of DEGs

We employed the TRRUST database to identify potential transcription factor targets of DEGs. Correlation analyses among DEGs were performed using the ggcorrplot and PerformanceAnalytics packages. The ssGSEA method was implemented to estimate infiltration levels of 22 immune cell types. Immune infiltration analysis and correlation analysis between DEGs and immune cell infiltration were conducted using the TIMER database, in conjunction with the geom_segment, geom_point, pheatmap, and ggplot2 packages.

Potential miRNA-mRNA interactions were predicted using the miRbase and TargetScan databases, while miRNA-lncRNA and lncRNA-mRNA interactions were explored through the starBase database. These predictions were integrated to construct a comprehensive regulatory network, which was subsequently visualized using the igraph and visNetwork packages.

For drug prediction, we utilized the DGIdb (Drug Gene Interaction Database) and CMap (Connectivity Map) databases combined with the enrichDGN function from the DOSE package. Results were visualized via barplot and dotplot. Web scraping was performed using the rvest package to retrieve relevant data. A drug-gene interaction network was constructed and analyzed using functions from the igraph package, enabling identification of potential drugs targeting the key genes.

### 2.7 Construction of renal tissue single-cell atlas and cellular clustering

All data analyses were performed using R (version 4.3.1; https://www.r-project.org/). Dataset integration and batch effect correction were conducted using the harmony package. Quality control was implemented via the Seurat package, which involved excluding cells expressing fewer than 500 genes, more than 7,000 genes, or containing mitochondrial genes accounting for >20% of total transcript counts. A total of 45,315 quality-filtered cells were retained for downstream analyses. Cellular clustering was performed using the FindClusters function at a resolution of 0.8, with cell identities annotated based on the top 5 marker genes. Dimensionality reduction was achieved through the RunTSNE function, and results were visualized using the ggplot2 package.

### 2.8 Visualization of differential gene expression patterns through density plots, bubble plots, and violin plots

We performed differential gene visualization using the ggplot2 package to generate density plots and bubble plots. The single-cell transcriptomic data from IMN patients were stratified into two disease progression groups (Stage II vs. Stages III/IV) according to pathological grading criteria. SPP1 expression levels across different pathological stages were statistically compared and visualized through violin plots created with ggplot2.

### 2.9 Identification of cellular population regulators

The gene regulatory network analysis was performed using pySCENIC following its three-step analytical pipeline. First, we inferred transcription factor (TF)-target gene co-expression modules through the GRNBoost2 algorithm, executing the pyscenic grn command to generate an adjacency matrix (sce.adj.csv) from the expression data (sce.loom) and human TF database. Second, we conducted TF-motif enrichment analysis using pyscenic ctx to identify direct target genes, resulting in regulons (sce.regulons.csv) where each regulon represents a TF and its directly regulated targets. The human genomic regulatory regions (hg38) were defined as ±10kb flanking transcription start sites. Finally, we scored regulon activity in individual cells via pyscenic aucell, which quantifies the enrichment of regulon genes in each cell’s expression profile.

### 2.10 Cell-cell interaction analysis

We performed cell-cell interaction analysis using the CellPhoneDB package in Python (version 3.13.2; https://www.python.org/), with particular focus on the interactions between SPP1-high PTCs and other cell populations that are mediated by inflammatory factors and chemokines.

### 2.11 Cell transfection

The SPP1 and NR2F1 silenced HK-2 cell lines were established via transfection with small interfering RNAs (siRNAs, Hycyte, China). HK-2 cells (TCH-C400, Hycyte, China) were transfected with siRNA, corresponding negative control (NC), and fluorescent control (FAM-NC) using Lipofectamine 2000 (Thermo Fisher Scientific, USA) according to the manufacturer’s (Hycyte, China) protocol. The optimized transfection conditions determined through preliminary experiments were: (1) cell confluence of 30-40%; (2) siRNA concentration of 30 nM; and (3) transfection duration of 32 hours.

The overexpression of the SPP1 gene in HK-2 cells was achieved via lentiviral infection (GenePharma, China), following the manufacturer’s instructions. The general procedure was as follows: When the density of the seeded cells reached 30%-40%, the experimental group (OE-SPP1) was supplemented with medium containing SPP1-overexpressing recombinant lentivirus and transfection-enhancing solution, while the control group (NC) was supplemented with medium containing empty vector lentivirus and transfection-enhancing solution. Twenty-four hours after infection, the medium was replaced with complete medium, and the cells were placed in a 37°C incubator for continued infection. After 72 hours of infection, the cells were cultured continuously in medium containing purinomycin for 7 days, thereby obtaining SPP1-overexpressing stable cell lines.

Transfection efficiency was validated by quantitative Real-Time PCR (qRT-PCR) and Western blot (WB) analyses.

### 2.12 RNA extraction, reverse transcription, and quantitative real-time PCR analysis

Total RNA was extracted from cells/tissues using the FastPure^®^ Cell/Tissue Total RNA Isolation Kit V2 (Vazyme, China), followed by cDNA synthesis with PrimeScript™ RT Master Mix (Takara, Japan) and subsequent qRT-PCR amplification using FastStart Essential DNA Green Master (Roche, Switzerland) according to manufacturers’ protocols, with β-actin as the endogenous control. Gene expression levels were quantified via the 2^−ΔΔCt^ method and statistically analyzed using GraphPad Prism 9.0, with data presented as mean ± SD and significance thresholds set at *P < 0.05, **P < 0.01, ***P < 0.001, and ****P < 0.0001 using unpaired t-tests for two-group comparisons or one-way ANOVA for multi-group analyses. The study evaluated key inflammatory markers—tumor necrosis factor-α (TNF-α), interleukin-1β (IL-1β), and interleukin-6 (IL-6)—along with fibrotic markers including transforming growth factor-β1 (TGF-β1), collagen type I (Col 1), vimentin (Vim), and fibronectin (FN) [19]. All primer sequences are detailed in **Supplementary Table 2** (GenSys Biotechnology, China).

### 2.13 Western blot analysis

The processed cells were washed twice with PBS (Solarbio, China) and lysed in RIPA buffer (Solarbio, China) containing 1% Protease and Phosphatase Inhibitor Cocktail (NCM Biotech, China) using ultrasonic cell disruption, followed by incubation on ice for 30 min and centrifugation at 10,000 rpm for 10 min to collect the protein supernatant. Protein concentration was determined by BCA assay (Beyotime, China). After quantification, loading buffer (Epizyme, China) was added at a 1:4 ratio, and samples were denatured at 100 °C for 10 min using a metal bath. Electrophoresis and transfer were performed using the Mini-PROTEAN Tetra system (Bio-Rad, USA) with the following parameters: 20V for 10 min, 80V for 20 min, and 120V for 60 min. Transfer conditions were optimized based on molecular weight: 400 mA for 30 min for proteins ≤100 kDa, and 220 mA for 90 min for Fibronectin. Membranes were blocked with blocking buffer (NCM Biotech, China) for 40 min, washed three times with TBST (Solarbio, China) (10 min per wash), and incubated with primary antibodies at 4°C overnight. After three additional TBST washes, membranes were incubated with HRP-conjugated secondary antibodies at room temperature for 1 h, washed again, and developed using ECL Ultra-sensitive substrate (Biosharp, China). Protein bands were visualized using a gel imaging system and quantified with ImageJ software. Detailed antibody information is provided in **Supplementary Table 3**.

### 2.14 Immunohistochemical and multiplex immunofluorescence assays

Renal tissue sections from IMN patients and controls were obtained from the Second Affiliated Hospital of Guangxi Medical University with donor/relative consent and ethical approval (2023KY-0715).

Immunohistochemistry was performed using a universal immunohistochemical kit (Proteintech, China) following the manufacturer’s protocol (antibody details in **Supplementary Table 4**). For multiplex immunofluorescence: Dewaxed tissue sections underwent antigen retrieval (EDTA solution, Solarbio, China) and BSA blocking (Sigma, USA). They were then sequentially incubated with primary antibodies (4°C, overnight) and fluorescent secondary antibodies (37°C, dark). For the second protein’s primary/secondary antibodies, incubation started at the BSA blocking step (all subsequent steps dark). Nuclei were stained with DAPI (Servicebio, China), sections mounted with anti-fluorescence quenching medium (Servicebio, China), and imaged via confocal microscope (antibody details in **Supplementary Table 5**).

## 3 Results

### 3.1 Identification of DEGs

**Figure 1:**
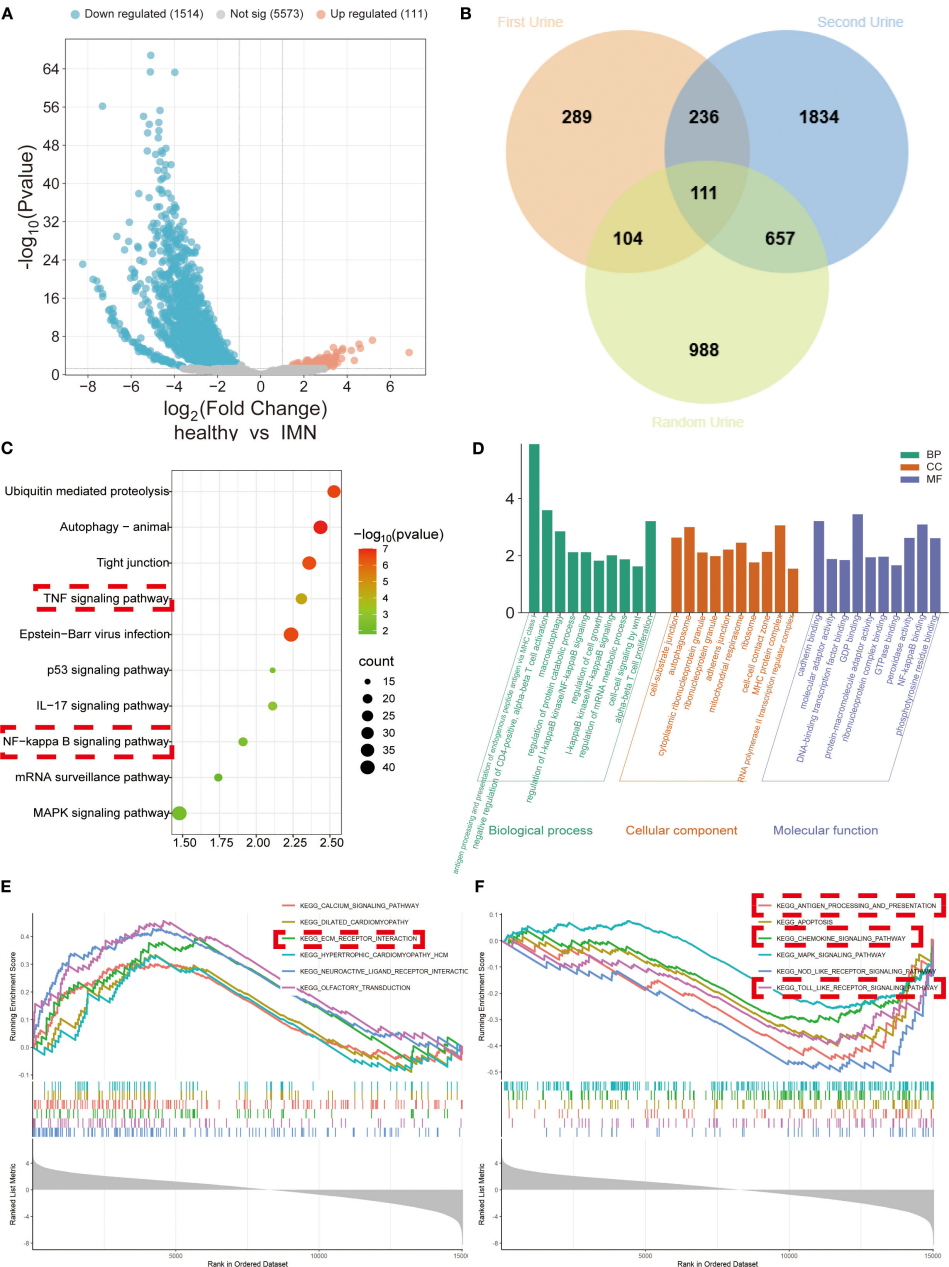
DEGs’ expression and functional enrichment analysis of urinary exfoliated cells from IMN patients versus healthy volunteers across three sample types. **(A)** Volcano plot displaying integrated DEGs from all three urine samples. **(B)** Venn diagram illustrating overlapping DEGs among sample types. **(C)** KEGG pathway analysis demonstrating significant associations of DEGs with inflammatory and fibrotic responses. **(D)** GO analysis categorizing DEGs into biological processes (BP), cellular components (CC), and molecular functions (MF). **(E, F)** GSEA of downregulated and upregulated gene sets, respectively.

We performed comparative analysis of three urine samples from 17 IMN patients and 17 healthy volunteers, integrating DEGs consistently identified across all three sample types with particular focus on genes showing elevated expression in all samples. Volcano plots and Venn diagrams of DEGs revealed 111 stably upregulated genes across different collection times (**Figures 1A, B**), demonstrating their robustness to sampling variability. KEGG, GO, and GSEA analyses indicated these DEGs were significantly enriched in inflammatory pathways (NF-κB, TNF, chemokine signaling, and Toll-like receptor pathways) and fibrotic processes (e.g., extracellular matrix receptor interactions) (**Figures 1C-F**). PPI network analysis of the 111 DEGs identified VIM as a central node interacting with multiple DEGs (**Supplementary Figures 1A, B**), further confirming the strong association between urinary cell DEGs and fibrotic mechanisms.

### 3.2 Identification of key factors including SPP1

**Figure 2:**
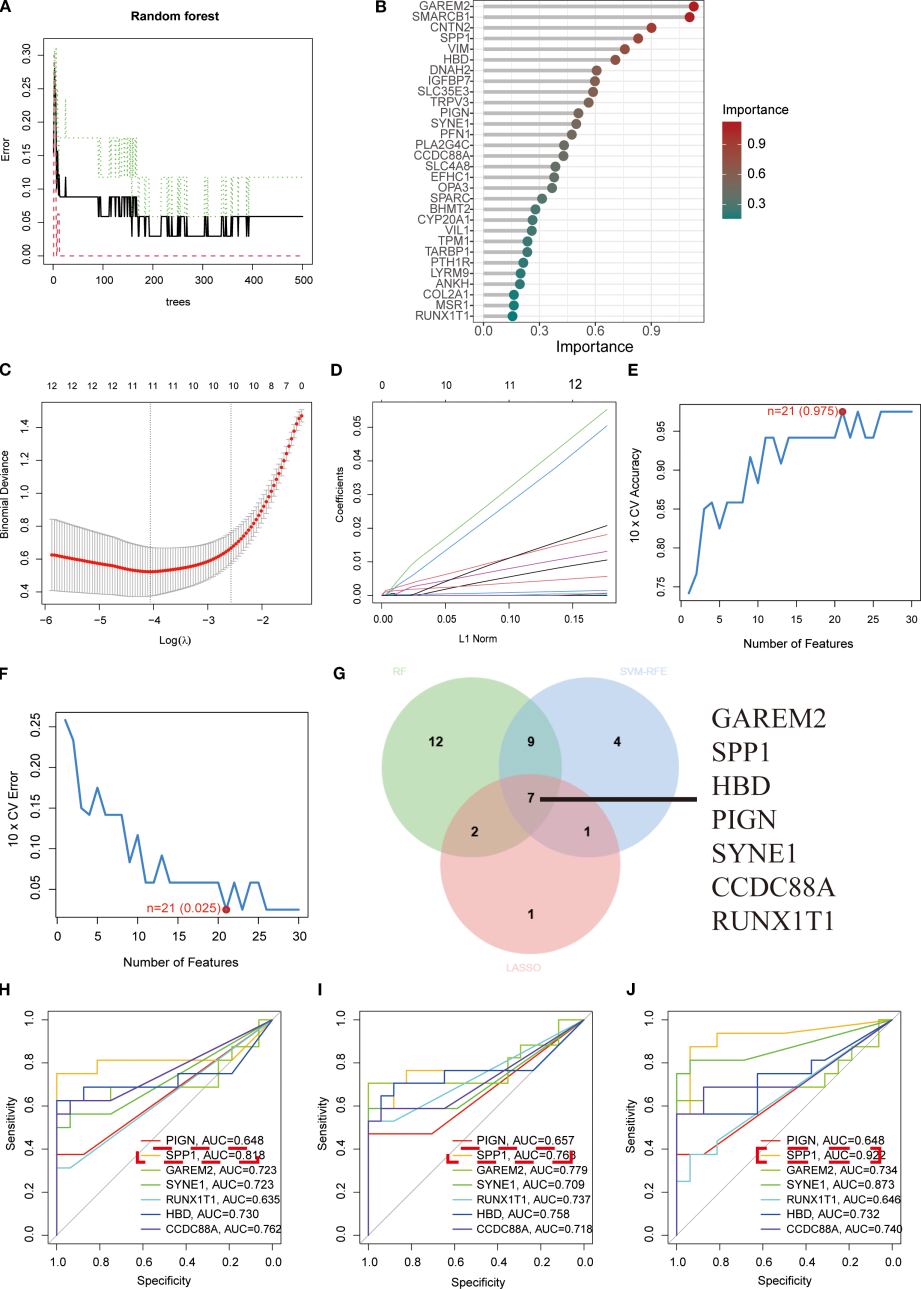
Screening of key biomarkers. **(A, B)** Feature importance identification using Random Forest algorithm. **(C, D)** Coefficient trajectory plot and cross-validation curve from LASSO logistic regression analysis. **(E, F)** Prediction accuracy and error variation curves for each gene in SVM algorithm. **(G)** Venn diagram displaying overlapping diagnostic biomarkers identified by all three algorithms. **(H-J)** ROC curves demonstrating diagnostic efficacy of the seven key biomarkers in first-void morning urine, second-void morning urine, and random urine samples, respectively, where higher AUC values indicate stronger correlations and better diagnostic performance.

We screened the 111 DEGs using three machine learning approaches—Random Forest, LASSO, and SVM (**Figures 2A-F**)—and identified overlapping key factors through Venn diagram analysis (**Figure 2G**). This integrated approach yielded seven pivotal biomarkers: GAREM2, SPP1, HBD, PIGN, SYNE1, CCDC88A, and RUNX1T1 (**Figure 2G**). ROC curve analysis demonstrated the diagnostic performance of these seven key factors across all three urine samples, revealing that SPP1 consistently exhibited superior diagnostic efficacy (**Figures 2H-J**).

### 3.3 Exploration of regulatory networks and diagnostic performance of key biomarkers

**Figure 3:**
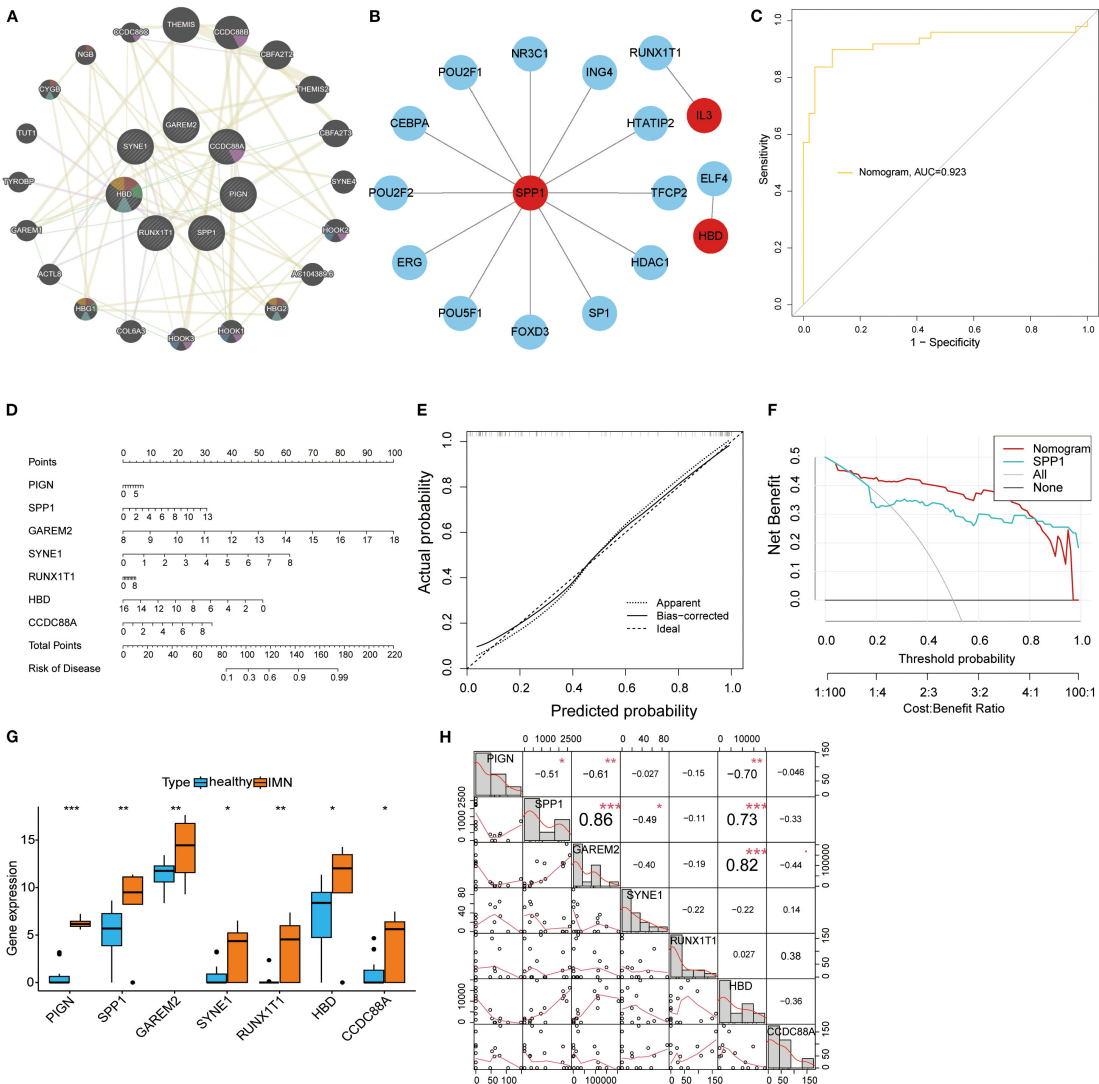
Transcriptional regulatory networks and diagnostic performance evaluation of key biomarkers. **(A)** PPI network of the seven key biomarkers. **(B)** Transcriptional regulatory networks of SPP1, RUNX1T1, and HBD identified through TRRUST database analysis. **(C)** ROC curve of the nomogram model, where higher AUC values indicate greater model reliability. **(D)** The nomogram visually demonstrates the association between key biomarkers and disease risk, with the length of each variable’s axis (i.e., scale markers) being proportional to its contribution to the outcome. **(E)** Calibration curve analysis of the nomogram model: Apparent represents the model’s predictive performance on the training set; Bias-corrected shows the relationship between predicted and actual probabilities after bias correction; Ideal indicates perfect alignment between predicted and actual probabilities. **(F)** Decision curve analysis for the SPP1 biomarker. **(G)** Box plots displaying expression levels of all key biomarkers. **(H)** Correlation analysis of DEGs: the lower left quadrant shows bivariate scatter plots with fitted lines, while the upper right quadrant displays correlation coefficients and significance levels (*P < 0.05, **P < 0.01, ***P < 0.001).

To investigate the functional roles of the seven key biomarkers, we analyzed their protein interaction networks, which revealed significant associations with developmental and inflammatory response proteins (**Figure 3A**). Using the TRRUST database, we identified NR3C1, POU2F1, CEBPA, POU2F2, ERG, POU5F1, FOXD3, SP1, HDAC1, TFCP2, HTATIP2, and ING4 as key transcription factors regulating SPP1, while RUNX1T1 and ELF4 were found to regulate IL-3 and HBD, respectively (**Figure 3B**). We constructed a nomogram model incorporating all seven biomarkers (PIGN, SPP1, GAREM2, SYNE1, RUNX1T1, HBD, and CCDC88A) that demonstrated strong diagnostic performance (AUC = 0.923, **Figure 3C**) and reliable risk prediction across a 0.1-0.99 probability range (**Figure 3D**). The model showed excellent calibration (**Figure 3E**) and provided superior net clinical benefit compared to single-marker approaches at threshold probabilities of 0.1-0.8 (**Figure 3F**). All seven biomarkers exhibited significantly higher expression levels in IMN patients than in controls when detected in second-void urine samples (**Figure 3G**), which lays a foundation for investigating the association between these biomarkers and IMN. Meanwhile, correlation analysis demonstrated positive correlations between SPP1 and GAREM2, SPP1 and HBD. In addition, PIGN was found to have negative correlations with SPP1, GAREM2, and HBD; SPP1 also showed a negative correlation with SYNE1 (**Figure 3H**).

### 3.4 Investigation of immune infiltration and immune cell correlations for key biomarkers

**Figure 4:**
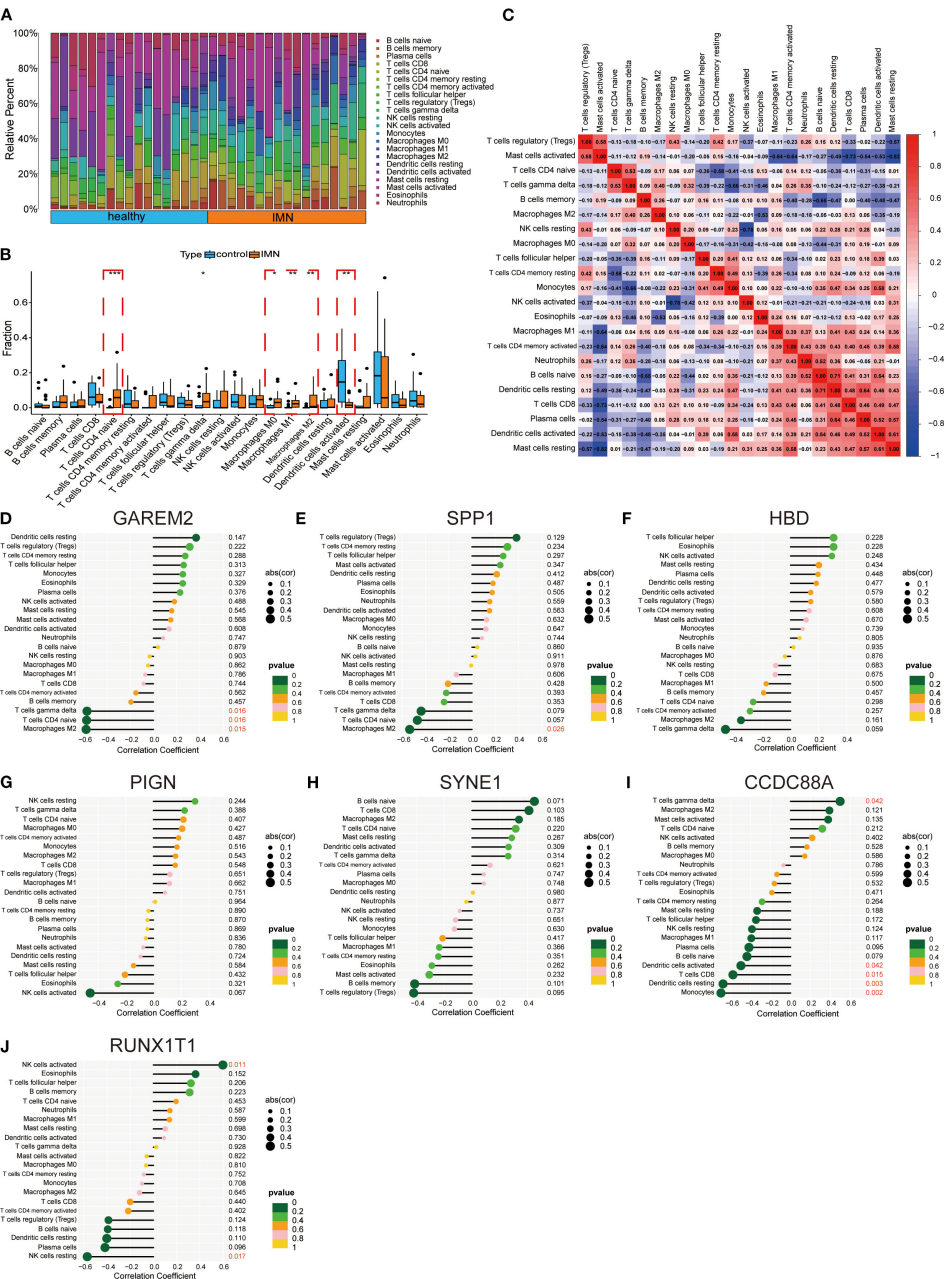
Immune infiltration and immune cell correlation analysis of key factors. **(A, B)** Immune cell infiltration between IMN and normal controls. **(C)** Heatmap of correlations between immune cells. **(D-J)** Lollipop plots showing correlations between hub genes and immune cells.

Immune infiltration analysis was performed using RNA-seq data from second-void morning urine samples. Comparative assessment between IMN patients and healthy controls revealed significantly higher proportions of macrophage subsets in IMN (**Figures 4A, B**). Heatmap analysis demonstrated significant positive correlations between plasma cells and dendritic cells resting, naïve B cells and dendritic cells resting, as well as activated dendritic cells and resting mast cells, while negative correlations were observed between regulatory T cells and resting mast cells, and M1 macrophages and activated mast cells (**Figure 4C**). Lollipop plots illustrated distinct immunocyte associations of the key biomarkers: GAREM2 showed negative correlations with γδ T cells, CD4^+^ naïve T cells, and M2 macrophages; SPP1 was inversely associated with M2 macrophages; CCDC88A positively correlated with γδ T cells but negatively with activated dendritic cells, CD8^+^ T cells, resting dendritic cells, and monocytes; RUNX1T1 exhibited positive correlation with activated NK cells but negative association with resting NK cells (**Figures 4D-J**). No significant correlations (*P > 0.05*) were found for the remaining biomarkers. These findings suggest that urinary DEGs may contribute to IMN pathogenesis by modulating the immune microenvironment, particularly through suppressing M2 macrophage infiltration and regulating T-cell subset homeostasis.

### 3.5 Regulatory factors and drug targets of key biomarkers

**Figure 5:**
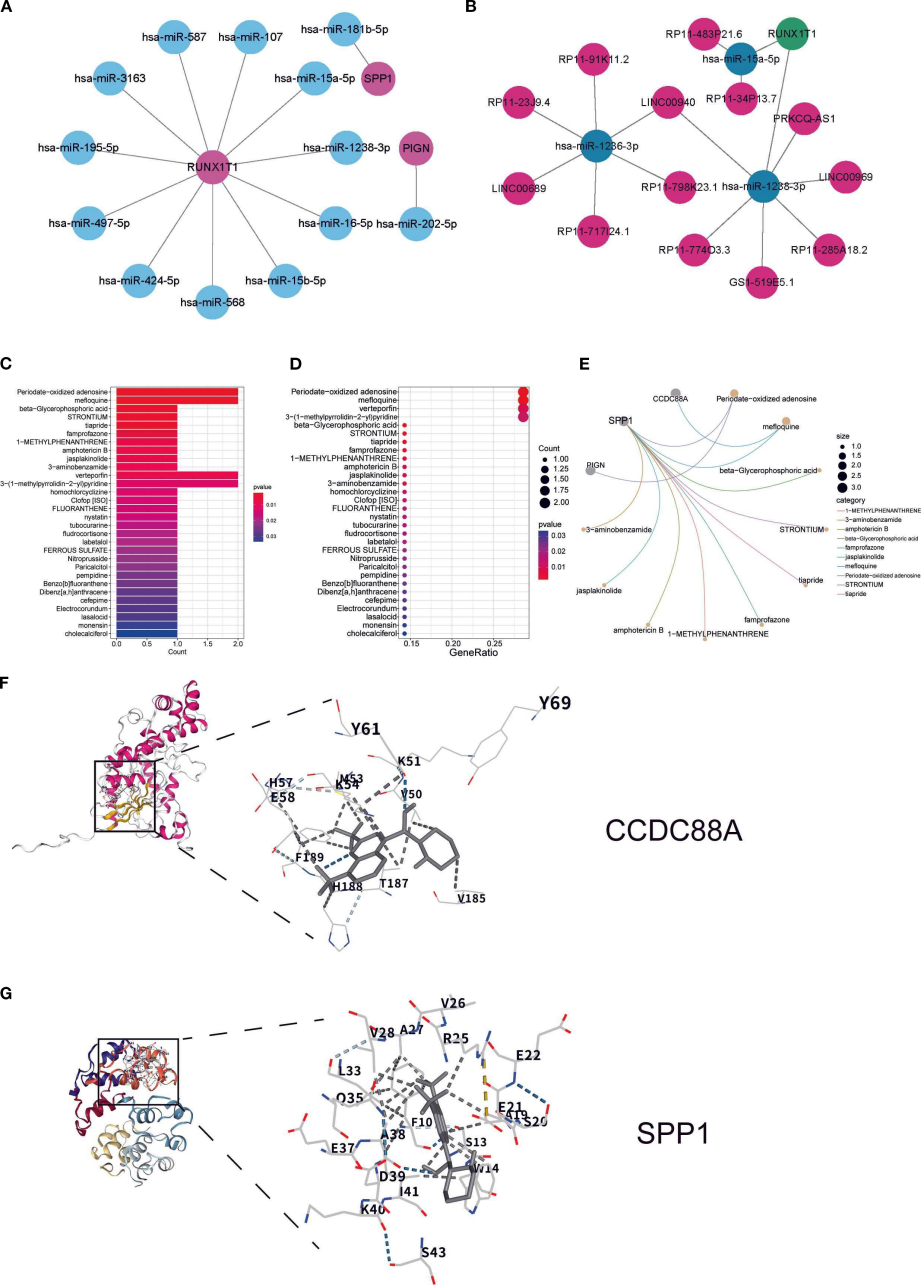
Regulatory factors and drug targets of key biomarkers: **(A)** miRNA regulatory network of key biomarkers; **(B)** lncRNA and ceRNA regulatory network of key biomarkers; **(C, D)** Box plot and bubble plot showing predicted drug binding sites; **(E)** Network diagram illustrating specific drug-biomarker interactions, where circular nodes represent key biomarkers and connecting lines indicate existing associations or interactions between drugs and biomarkers. **(F, G)** Molecular docking analysis showing the binding sites of CCDC88A and SPP1 with mefloquine.

The miRNA regulatory network revealed 11 miRNAs targeting RUNX1T1, 1 miRNA targeting SPP1, and 1 miRNA targeting PIGN (**Figure 5A**). The lncRNA-ceRNA network analysis identified only RUNX1T1 as being regulated by hsa-miR-1238-3p and hsa-miR-15a-5p (**Figure 5B**). Drug prediction analysis identified compounds with binding potential to key biomarkers: periodate-oxidized adenosine, mefloquine, verteporfin, and 3-(1-methylpyrrolidin-2-yl) pyridine showed binding capacity with two DEGs each (**Figures 5C, D**). Notably, SPP1 demonstrated binding potential with nearly all analyzed drugs, suggesting strong therapeutic promise (**Figure 5E**). Mefloquine, an established clinical drug with recently reported immunomodulatory properties (21), was further investigated through molecular docking experiments. The results confirmed stable binding conformations between mefloquine and both SPP1/CCDC88A proteins (**Figures 5F, G**), with binding energies <-5 kcal/mol, indicating high affinity and potential as therapeutic targets.

### 3.6 Construction of a single-cell atlas of IMN renal tissue

**Figure 6:**
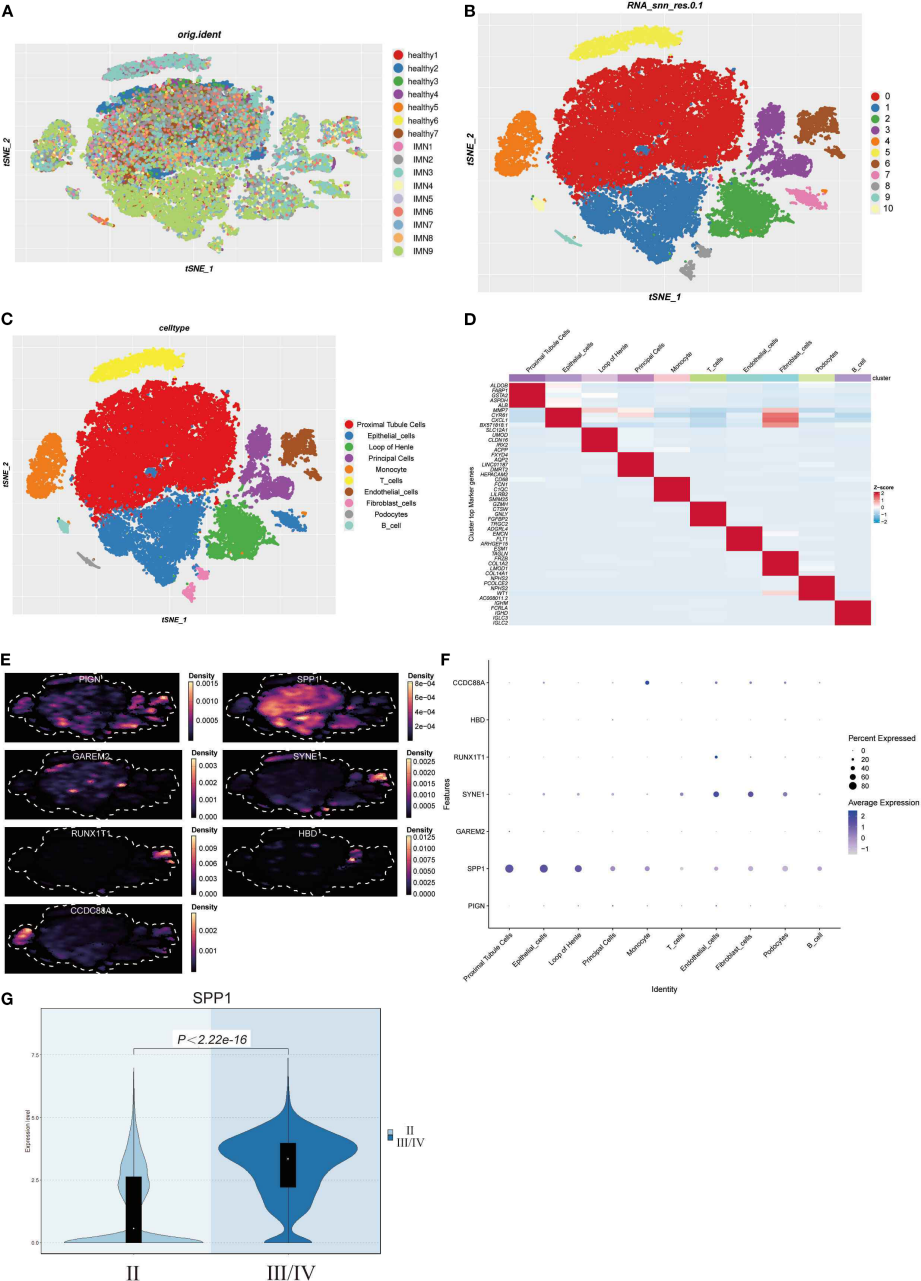
Construction of IMN single-cell atlas and cellular localization of differentially expressed genes: **(A)** Integration of scRNA-seq data from renal tissues of 9 IMN patients and 7 healthy controls; **(B, C)** Cell clustering into 10 distinct populations based on marker gene expression; **(D)** Marker genes of each cell cluster are shown as a heatmap, with color intensity indicating their relative expression; **(E, F)** Density plots and bubble charts visualizing distribution patterns of differentially expressed genes across cell clusters - in density plots, color intensity correlates positively with cellular distribution density (darker shades indicate higher cell density), while in bubble charts, bubble size is proportional to cell numbers (larger bubbles represent greater cell quantities); **(G)** Violin plot analysis demonstrating significantly elevated SPP1 expression in stage III/IV pathological samples compared to earlier stages.

Integrated analysis of scRNA-seq data from 9 IMN patients and 7 healthy controls (**Figure 6A**) after quality control (**Supplementary Figure 2**) revealed 10 distinct cell clusters through principal component analysis of variably expressed genes: proximal tubular cells, epithelial cells, loop of Henle cells, principal cells, monocytes, T cells, endothelial cells, fibroblasts, podocytes, and B cells (**Figures 6B, C**). Cluster-specific marker genes were visualized by heatmap, with color intensity reflecting relative expression levels (**Figure 6D**). Density plots and bubble charts demonstrated cellular expression patterns of 7 differentially expressed genes, where darker hues indicated higher expression (**Figures 6E, F**). SPP1 showed ubiquitous expression across all clusters with peak abundance in proximal tubular cells (**Figure 6F**), suggesting its potential involvement in IMN pathogenesis. Stratification by pathological stage (II vs. III/IV) demonstrated upregulated SPP1 expression with broader distribution in advanced-stage samples (**Figure 6G**), indicating a potential correlation between SPP1 expression levels and disease progression, which may implicate SPP1 as a key contributor to IMN advancement.

### 3.7 Exploration of the functions and key regulatory factors of SPP1-high expressing PTCs populations

**Figure 7:**
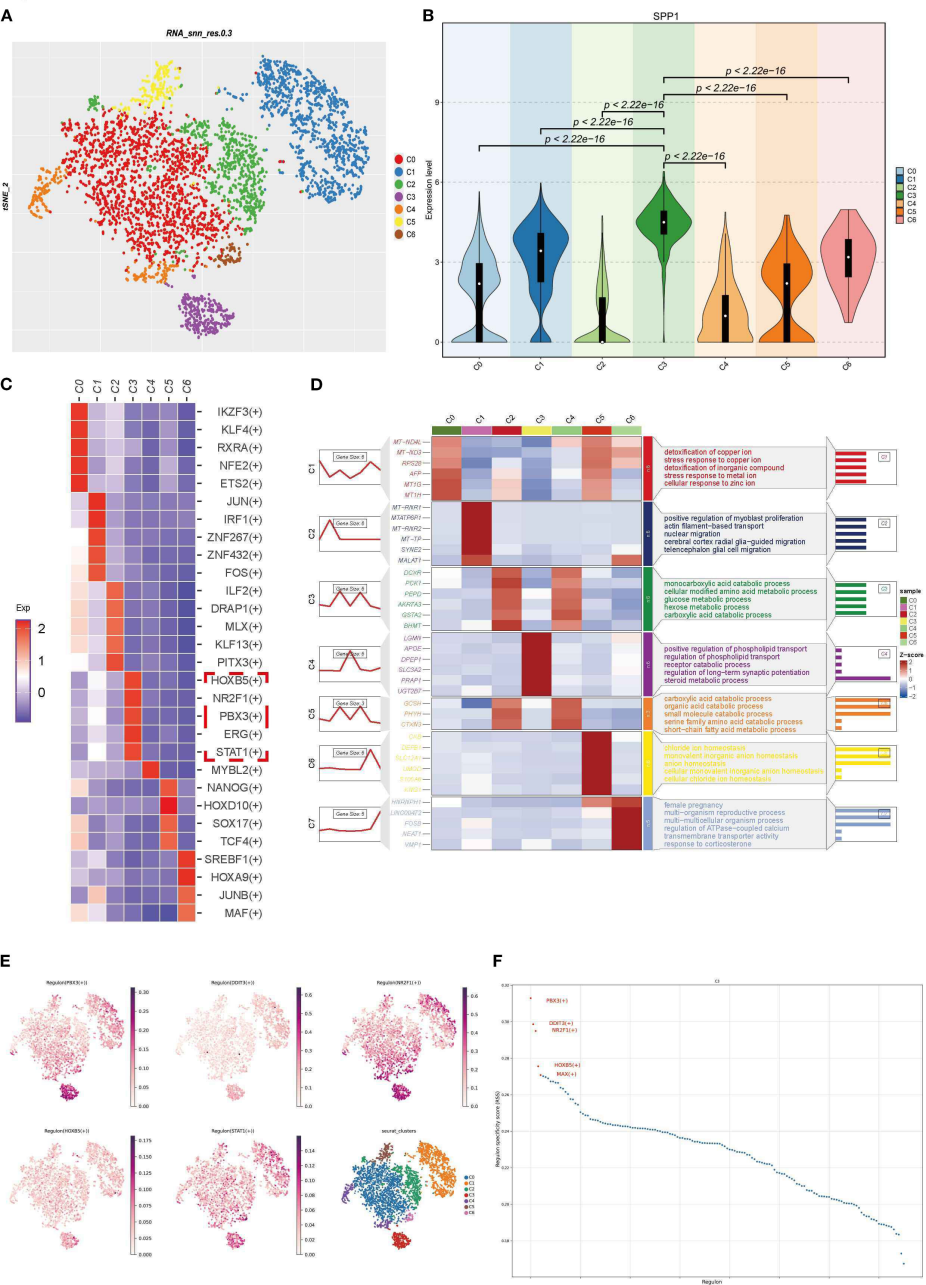
Functional exploration and transcriptional regulatory factor analysis of SPP1-high expressing PTCs clusters: **(A)** PTCs were reclassified into 7 clusters with a resolution of 0.1; **(B)** Violin plot showing high SPP1 expression in cluster C3; **(C)** Heatmap of transcription factors with high specificity for each cluster, calculated by RSS; **(D)** Heatmap visualizing characteristic marker gene expression and corresponding GO enrichment analysis results; **(E)** TSNE plot displaying transcription factor distribution, where each point represents a cell. The color intensity indicates the activity level of the corresponding transcription regulator (darker color = higher activity), enabling observation of activity differences across cells; **(F)** Scatter line plot with abscissa “Regulon” (different transcription regulators) and ordinate “Regulon specificity score” (reflecting regulator specificity—higher scores indicate stronger unique activity in specific cell types/states).

We reclassified PTCs into 7 clusters and found that SPP1 was highly expressed in cluster C3 (**Figures 7A, B**). Using Regulon Specificity Score (RSS) analysis, we generated a heatmap of key regulatory factors for this cluster, as shown in **Figure 7C**. HOXB5, NR2F1, PBX3, ERG, and STAT1 were identified as critical transcription factors for cluster C3. Enrichment analysis of the top 3–6 DEGs in each subgroup was performed: the left line chart depicts the expression patterns of DEGs, while the right panel shows GO enrichment results using these DEGs, reflecting biological process pathways and functional enrichment of DEGs in each cluster (**Figure 7D**). Notably, cluster C3 (labeled as cluster C4 in the figure) was associated with positive regulation of phospholipid transport and steroid metabolism (**Figure 7D**). TSNE visualization of transcription factor activity showed that NR2F1 and PBX3 had the highest activity in cluster C3, indicating their dominant regulatory role in this cluster (**Figure 7E**). Line charts suggested that PBX3, DDIT3, and NR2F1 might exhibit more specific regulatory effects in cluster C3 (**Figure 7F**). Intriguingly, NR2F1 belongs to the same nuclear receptor superfamily as NR3C1 (**Figure 3B**), implying that nuclear receptor superfamily members may serve as key regulators of SPP1. The regulatory role of NR2F1 in SPP1 expression warrants further investigation.

### 3.8 Cell-cell interactions in cluster C3

**Figure 8:**
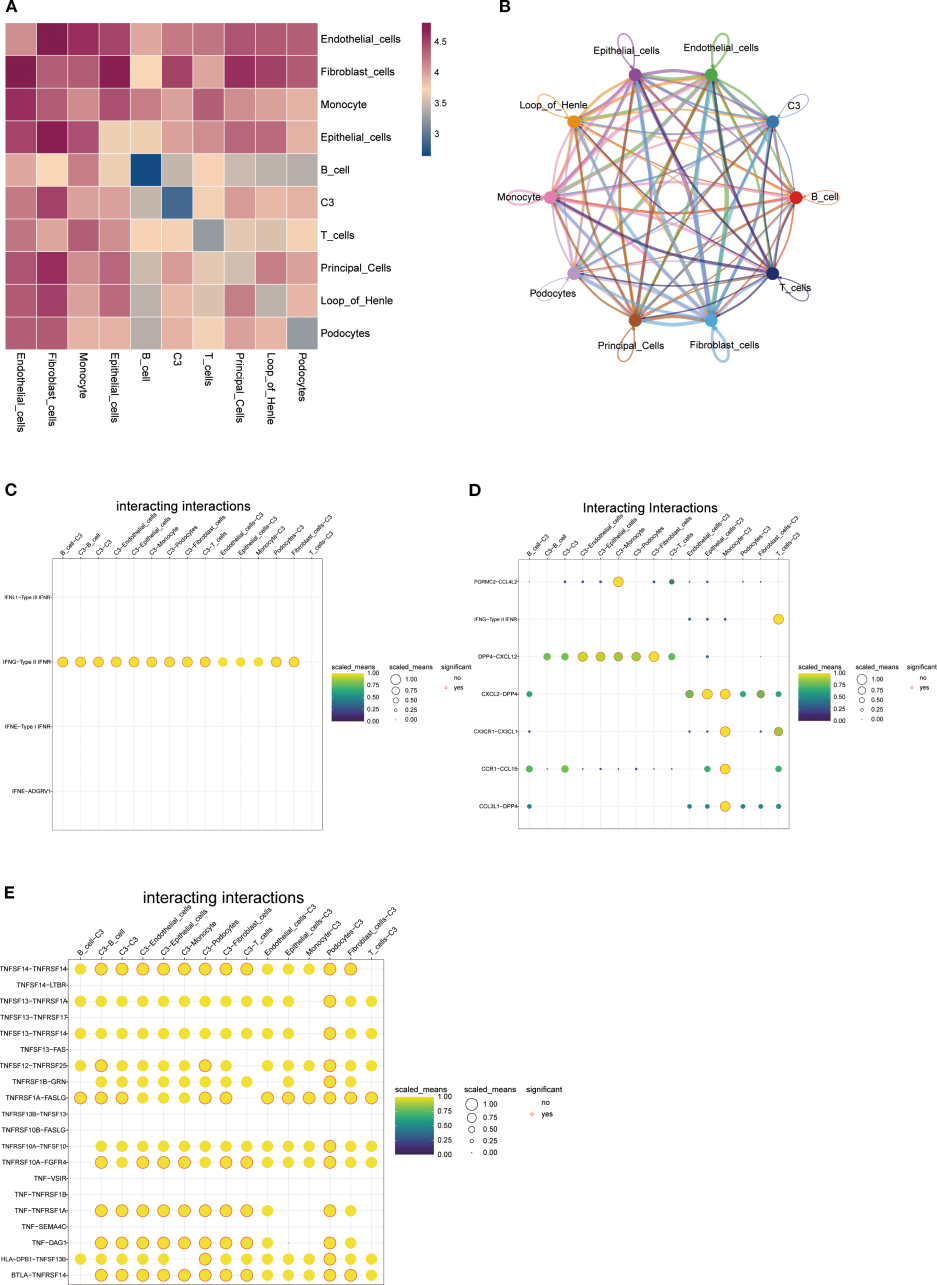
Cell-cell interaction networks: **(A)** Heatmap of intercellular interactions where deep red indicates stronger associations and blue denotes weaker correlations; **(B)** Network diagram displaying interaction relationships between cell clusters (colored nodes represent distinct cell types, connecting lines indicate significant interactions-greater line complexity reflects richer intercellular crosstalk); **(C-E)** Bubble plots demonstrating C3 cell cluster interactions via **(C)** interferon family factors, **(D)** CC-chemokine subfamily factors, and **(E)** tumor necrosis factor superfamily members (yellow-shaded bubbles indicate higher normalized values, red-circled solid dots denote statistical significance).

Through heatmap analysis and network visualization, we observed significant interaction characteristics between different cell populations (**Figures 8A, B**). Notably, the interaction network between cluster C3 and fibroblasts exhibited the densest connections and a significantly higher signal communication intensity compared to other cell populations, suggesting that their close interaction may play a critical role in renal tissue fibrosis (**Figure 8A**). Given SPP1’s potential association with inflammatory responses in IMN, we focused on three key cytokine families—the interferon (IFN) family, chemokine CC subfamily (CCL), and tumor necrosis factor (TNF) family—to systematically explore their intercellular networks. Cluster C3 signals to other cell populations by secreting type II interferon (IFN-γ) and binding to its receptor (IFNGR) (**Figure 8C**). Furthermore, cluster C3 connects with mononuclear macrophages via CCL members, potentially regulating their migration and activation (**Figure 8D**). Additionally, the TNF family mediates interactions between cluster C3 and other cells through TNF-TNFR signaling, dynamically regulating the immune microenvironment (**Figure 8E**). These findings highlight cluster C3’s central role in intercellular communication and its multidimensional regulatory mechanisms.

### 3.9 Immunohistochemistry and renal tissue qRT-PCR validation of SPP1 expression in IMN

**Figure 9:**
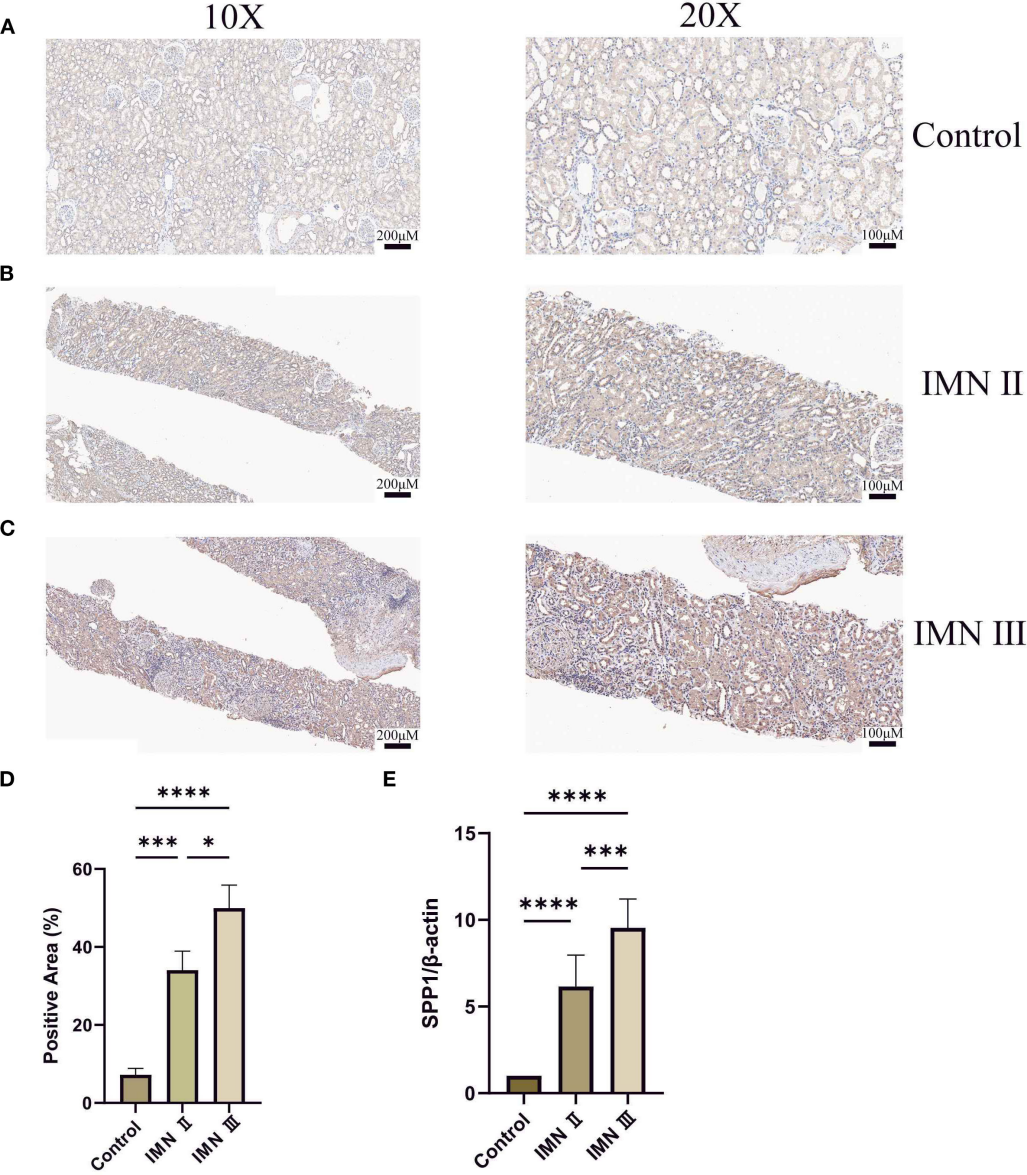
Detection of SPP1 expression in renal tissues by immunohistochemistry and qRT-PCR: **(A-C)** Immunohistochemical staining of SPP1 in control, stage II IMN, and stage III IMN renal tissues, respectively. Darker coloration indicates higher SPP1 expression levels. **(D)** Quantitative immunohistochemical analysis of the control, stage II IMN, and stage III IMN renal tissues. **(E)** qRT-PCR analysis of SPP1 expression in adjacent normal renal tissues, stage II IMN, and stage III IMN renal tissues. (*P < 0.05, **P < 0.01, ***P < 0.001, ****P < 0.0001).

Immunohistochemical analysis revealed that SPP1 expression was highest in stage III IMN renal tissues, followed by stage II, while the control group showed the lowest expression level (**Figures 9A-C**). Quantitative analysis of immunohistochemistry confirmed that these results were statistically significant (**Figure 9D**). Consistently, qRT-PCR analysis of IMN renal tissues and adjacent normal renal tissues demonstrated that SPP1 expression positively correlated with IMN pathological severity (**Figure 9E**). This finding aligns closely with prior bioinformatics analyses, suggesting that SPP1 may be associated with IMN disease progression.

### 3.10 Validation of SPP1 and NR2F1 knockdown efficiency and expression of inflammatory and fibrotic factors

**Figure 10:**
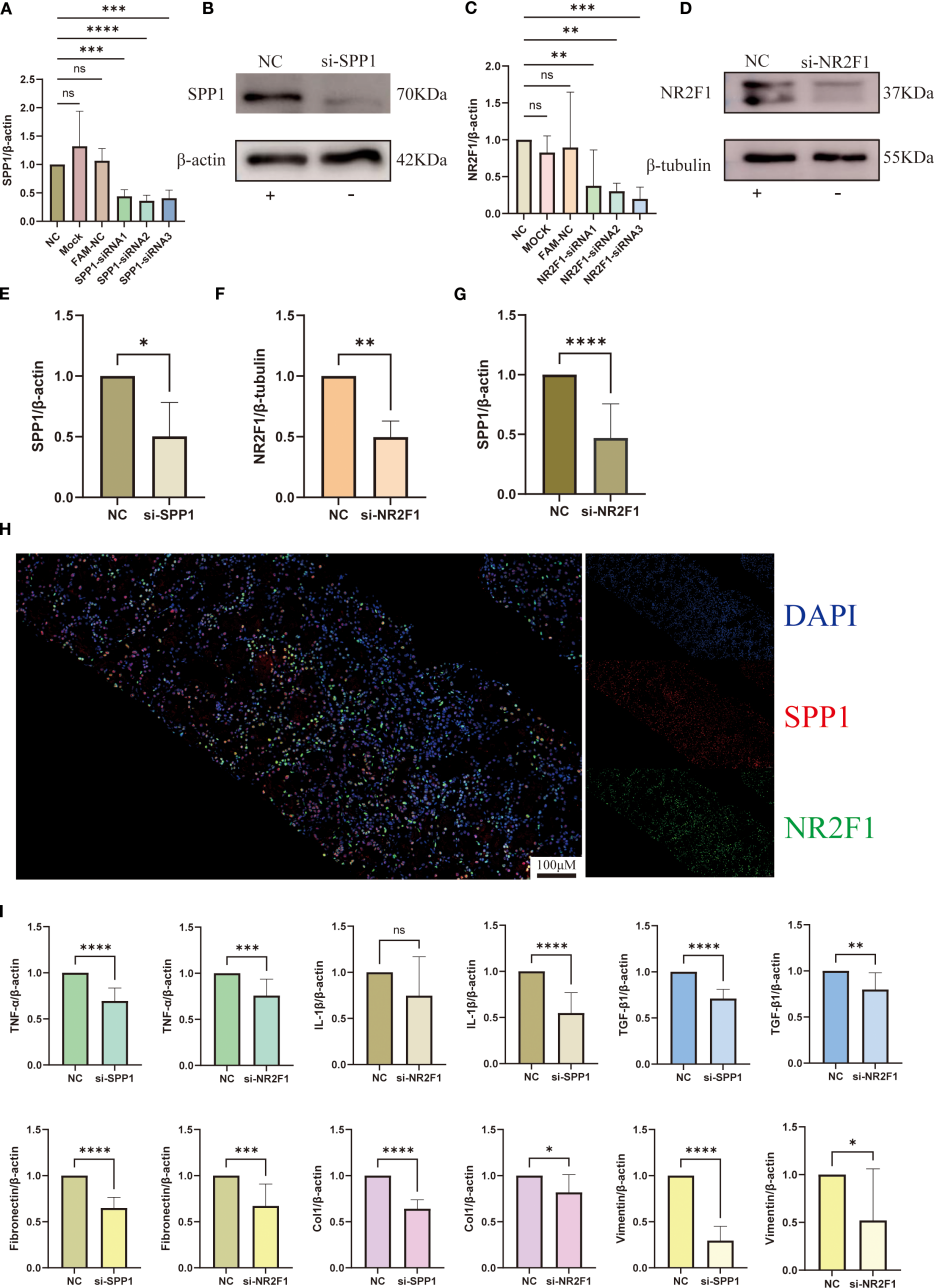
Interaction between SPP1 and NR2F1 and their regulatory mechanisms in inflammation and fibrosis: **(A-F)** Silencing efficiency of SPP1 and NR2F1 was validated by qRT-PCR and WB; **(G)** Altered SPP1 expression levels in si-NR2F1 group; **(H)** Dual-color immunofluorescence revealing intracellular spatial co-localization of SPP1 and NR2F1; **(I)** Expression profiles of inflammatory and fibrotic markers in si-SPP1 and si-NR2F1 group. (*P < 0.05, **P < 0.01, ***P < 0.001, ****P < 0.0001).

We performed siRNA transfection experiments in HK-2 cells, using siRNA carrying scrambled sequences as the NC group. qRT-PCR results demonstrated that SPP1-siRNA2 and NR2F1-siRNA3 exhibited the optimal gene knockdown efficiency, while neither the transfection reagent control (MOCK) or the FAM-NC showed significant effects on SPP1/NR2F1 mRNA expression (**Figures 10A, C**). WB analysis confirmed successful knockdown of SPP1 and NR2F1 at the protein level (**Figures 10B, D, E, F**).

qRT-PCR analysis revealed significantly reduced SPP1 expression in the NR2F1 knockdown group (si-NR2F1), providing cellular-level evidence for NR2F1’s regulatory role on SPP1 (**Figure 10G**). Furthermore, immunofluorescence results demonstrated co-localization of NR2F1 and SPP1 in cells, offering histological support for the hypothesis that NR2F1 may serve as a regulatory factor for SPP1 (**Figure 10H**).

To further investigate the relationship between SPP1 and inflammatory/fibrotic factors, we measured their expression levels by qRT-PCR. Results showed that in the SPP1 knockdown group (si-SPP1), expression of inflammatory factors (TNF-α, IL-1β) and fibrotic markers (TGF-β1, Col 1, Vim, FN) was significantly reduced (**Figure 10I**). In the si-NR2F1 group, expression of TNF-α, TGF-β1, Col 1, Vim and FN also decreased, while IL-1β expression remained unchanged (**Figure 10I**). Notably, although the expression levels of these inflammatory and fibrotic factors in the si-NR2F1 group were lower than controls, they were consistently higher than those in the si-SPP1 group.

### 3.11 Validation of inflammatory and fibrotic markers at histological and protein levels

**Figure 11:**
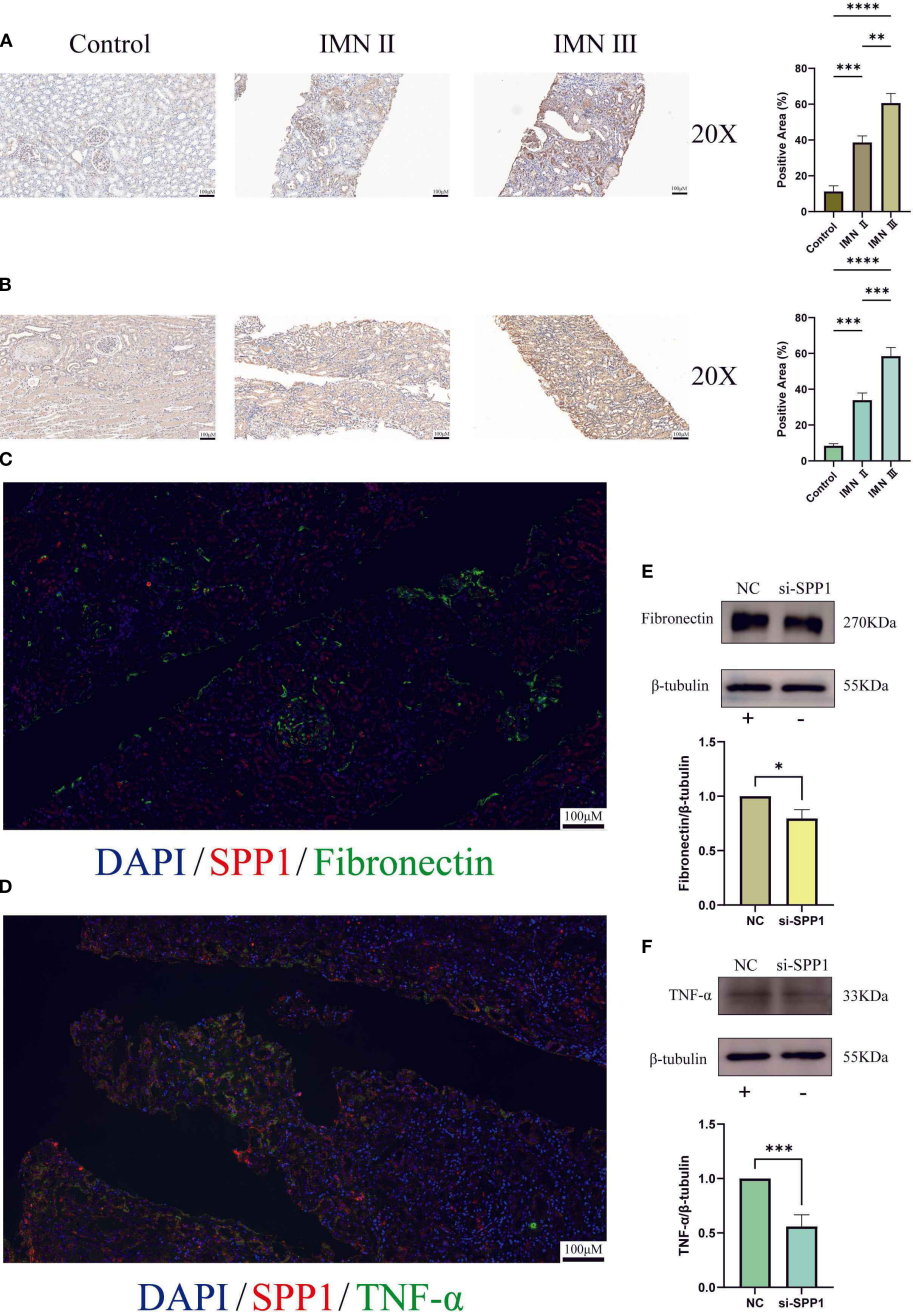
Immunohistochemistry, multicolor immunofluorescence, and construction of an SPP1-silenced cell line were performed to investigate the relationships between SPP1 and both Fibronectin as well as TNF-α: **(A)** Immunohistochemical expression profiles of Fibronectin in control, stage II IMN, and stage III IMN renal tissues; **(B)** Immunohistochemical expression patterns of TNF-α in control, stage II IMN, and stage III IMN renal tissues; **(C)** Dual-color immunofluorescence co-localization analysis of SPP1 and Fibronectin, revealing their spatial distribution correlation; **(D)** Dual-color immunofluorescence co-localization analysis of SPP1 and TNF-α, demonstrating their spatial association; **(E)** Effect of SPP1 silencing on Fibronectin expression levels; **(F)** Effect of SPP1 silencing on TNF-α expression levels. (*P < 0.05, **P < 0.01, ***P < 0.001, ****P < 0.0001).

Given that TNF-α and FN are hallmark factors of inflammation and fibrosis respectively, we selected these two markers to further validate SPP1’s regulatory effects on their expression. Immunohistochemical analysis revealed that both FN and TNF-α exhibited the highest expression levels in stage III IMN renal tissues, followed by stage II IMN, while control tissues showed minimal expression (**Figures 11A, B**). And the quantitative analysis of immunohistochemistry confirmed that these results were statistically significant. This expression pattern closely mirrored SPP1’s distribution in renal tissues. Immunofluorescence experiments further demonstrated significant co-localization between SPP1 and TNF-α, as well as between SPP1 and FN in tissues, providing histological evidence supporting SPP1’s potential role as a regulatory factor for both TNF-α and FN (**Figures 11C, D**). WB analysis confirmed that SPP1 knockdown reduced protein expression levels of both FN and TNF-α, offering additional protein-level validation of SPP1’s regulatory function (**Figures 11E, F**).

**Figure 12:**
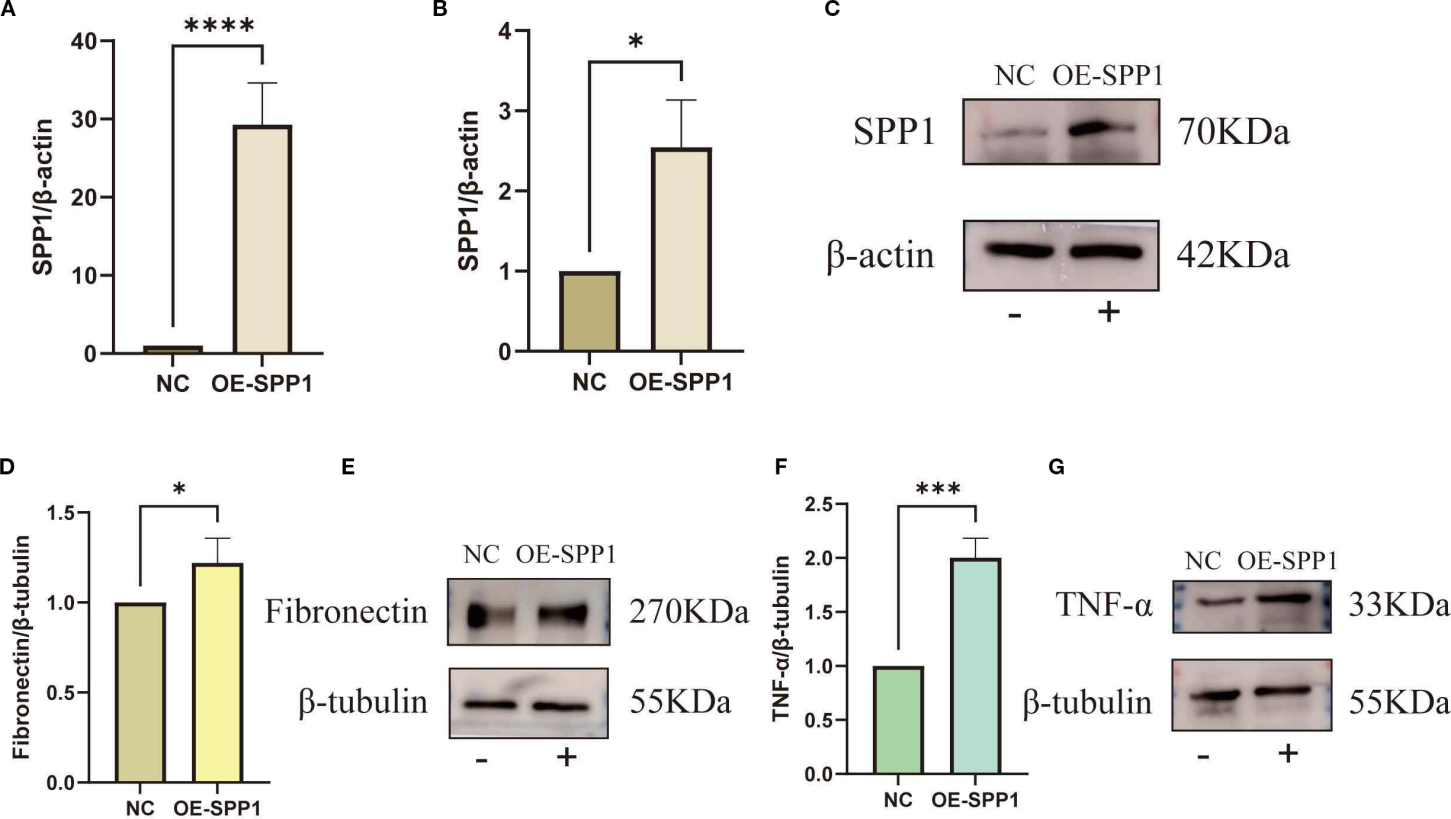
Overexpression of SPP1 to investigate the relationships between SPP1 and both Fibronectin as well as TNF-α: **(A-C)** Overexpression efficiency of SPP1 was validated by qRT-PCR and WB; **(D-E)** WB assay showing the expression levels of TNF-α in the OE-SPP1 group; **(F, G)** WB assay showing the expression levels of FN in the OE-SPP1 group. (*P < 0.05, **P < 0.01, ***P < 0.001, ****P < 0.0001).

Furthermore, we successfully constructed an SPP1-overexpressing cell line (**Figures 12A, C**). Experimental results showed that following SPP1 overexpression, the expression levels of the inflammatory marker TNF-α and the fibrosis-related factor FN were both significantly increased (**Figures 12D-G**). Collectively, these findings demonstrate that SPP1 exerts a crucial regulatory role in both inflammatory responses and fibrotic processes, while also suggesting its potential as a promising therapeutic target for IMN.

## 4 Discussion

Renal biopsy remains the gold standard for diagnosing kidney diseases, yet it only provides static pathological information and cannot dynamically assess disease progression or prognosis (3, 22, 23). Therefore, complementary diagnostic indicators are needed. Microscopic analysis of urinary sediment cells represents one of the oldest diagnostic tools in nephrology and the most common clinical application of urinary exfoliated cells (24). Studies have revealed that urinary exfoliated cells contain not only erythrocytes, microorganisms, and immune cells but also various renal-origin cells, including tubular epithelial cells (25), podocytes (26), and even undifferentiated renal progenitor cells (27). This demonstrates the significant potential of urinary exfoliated cells for both nephrology research and clinical applications. In our study, we observed consistently high and stable expression of SPP1 in urinary samples from IMN patients across three collections.

Biomarkers, defined as objectively measurable indicators of normal biological processes, pathological processes, or responses to therapeutic interventions, play crucial roles in disease diagnosis, prognosis evaluation, treatment monitoring, and drug development (28, 29). Emerging analytical approaches like network pharmacology and molecular docking have enabled systematic exploration of targeted drugs from molecular to pathway levels, gaining wide application in drug discovery research (30, 31). Our findings demonstrate that renal tubular SPP1 exhibits excellent diagnostic performance in serial urinary analyses and possesses multiple targetable binding sites, strongly supporting its potential as a biomarker for IMN.

PTCs play a pivotal role in maintaining renal reabsorption, secretion, and excretion functions, as well as water-electrolyte homeostasis (32). Emerging evidence indicates that PTCs injury can actively drive disease progression and even serve as a key contributor to renal dysfunction (33). During inflammatory responses, damaged PTCs release various inflammatory cytokines (e.g., TNF-α, IL-1β) and recruit immune cells through chemokine expression (e.g., MCP-1), thereby exacerbating local inflammation (34). Moreover, injured PTCs activate critical signaling pathways like NF-κB, further promoting inflammatory cytokine release and establishing a self-perpetuating inflammatory cycle (35). In fibrotic processes, damaged PTCs may undergo epithelial-mesenchymal transition into fibroblasts, facilitating excessive extracellular matrix (ECM) deposition and ultimately leading to renal fibrosis (36). Our scRNA-seq analysis of IMN renal tissues revealed predominant SPP1 expression in PTCs, with expression levels strongly correlating with IMN pathological severity. Notably, SPP1^+^ PTC populations occupied central positions in renal cellular interaction networks and demonstrated close associations with inflammatory/fibrotic responses.

Renal inflammation initially represents a physiological response to injury (37). However, persistent inflammation underlies the pathogenesis of numerous renal diseases, promoting fibrotic processes that drive chronic nephritis progression, functional decline, and eventual end-stage renal disease (38, 39). Conversely, ECM remodeling during fibrosis reciprocally fuels inflammation by facilitating immune cell migration and immunological synapse formation (40). As an autoimmune disorder, IMN pathogenesis fundamentally involves dysregulated immune responses (19), with inflammation and fibrosis constituting two core pathological processes driving disease progression (41). These interconnected mechanisms form a vicious cycle that significantly contributes to renal function loss (42, 43). Our findings demonstrate parallel elevation of TNF-α and FN expression with IMN progression, highlighting the therapeutic imperative of targeting inflammation-fibrosis crosstalk to mitigate disease advancement.

SPP1, a multifunctional glycosylated phosphoprotein, plays significant roles in diverse physiological and pathological processes (14, 44). It contains multiple functional domains that mediate biological effects through binding to cell surface receptors. Its expression and function are regulated by various factors, including transcriptional activity, epigenetic modifications, extracellular signals, and microenvironmental cues (45). In immune regulation, SPP1 participates in inflammatory responses by modulating the activation and migration of immune cells (e.g., macrophages and T cells) (46). Renal fibrosis studies have identified SPP1 as a key effector that promotes fibroblast-to-myofibroblast differentiation via the TGF-β/Smad signaling pathway (17, 47). Our study demonstrated that SPP1 knockdown in IMN significantly reduced the expression of TNF-α, IL-1β, TGF-β1, Col 1, Vim, and FN. And by constructing an SPP1-overexpressing cell line, we observed that the expression levels of TNF-α and FN increased in parallel with elevated SPP1 expression. Immunofluorescence further revealed close spatial associations between SPP1 and both TNF-α/FN at the tissue level, corroborating SPP1’s regulatory role in inflammation and fibrosis.

Notably, the downregulation of inflammatory and fibrotic factors was more moderate in the si-NR2F1 group compared to the si-SPP1 group. We hypothesize that NR2F1 knockdown may indirectly modulate these factors through SPP1 regulation. This finding not only reinforces SPP1’s central regulatory position in inflammation and fibrosis but also elucidates a potential molecular mechanism whereby NR2F1 exerts indirect effects via SPP1, providing a theoretical foundation for targeting SPP1 to mitigate fibrotic and inflammatory responses in IMN.

However, this study has several limitations. First, the urinary exfoliated cell RNA-seq analysis included only 17 IMN patients and 17 healthy controls with triplicate urine samples—a relatively small cohort that warrants expansion to validate SPP1’s reliability as an IMN biomarker. Second, although histological and *in vitro* cellular experiments confirmed SPP1’s regulatory effects, animal models are needed to comprehensively characterize its functional roles in inflammation and fibrosis. Finally, the precise molecular pathways through which SPP1 regulates these processes remain unexplored. Further mechanistic studies are essential to solidify SPP1’s potential as a therapeutic target.

## 5 Conclusions

The SPP1 factor in proximal tubular epithelial cells is closely associated with IMN disease progression, serving as a key biomarker and regulatory factor in both inflammatory and fibrotic processes. Targeted inhibition of SPP1 expression significantly reduces the levels of inflammation and fibrosis related factors, thereby effectively mitigating disease progression. These findings highlight SPP1 as a promising therapeutic target for IMN, offering a potential novel strategy for clinical intervention.

## Data Availability

The scRNA-seq raw data used in this study are deposited in the GEO database under accession number GSE241302, GSE171458, GSE131685. These data are publicly available for research purposes. In addition, the RNA-seq expression matrices of urine exfoliated cells from three times have been uploaded to **Supplementary Table 6**, where samples starting with "PL04" represent healthy volunteers, and the remaining samples represent those from IMN patients.

## Supplementary material

Supplementary Figure 1 PPI network of co-upregulated genes across three urine samples.

Supplementary Figure 2 scRNA-seq quality control plots of 9 IMN renal tissues and 7 normal renal tissues.

Supplementary Table 1 Clinical information table for patients and healthy volunteers.

Supplementary Table 2 Table of primer sequences used in this study.

Supplementary Table 3 Detailed table of antibodies used in Western Blot.

Supplementary Table 4 Detailed table of antibodies used in Immunohistochemistry.

Supplementary Table 5 Detailed table of antibodies used in Immunofluorescence.

Supplementary Table 6 RNA-seq Expression Matrix of Urinary Exfoliated Cells.

## References

[1] Ronco P Debiec H. Molecular pathogenesis of membranous nephropathy. Annu Rev Pathol. (2020) 15:287–313. doi: 10.1146/annurev-pathol-020117-043811, PMID: 31622560

[2] Meyer-Schwesinger C Lambeau G Stahl RAK. Thrombospondin type-1 domain-containing 7A in idiopathic membranous nephropathy. N Engl J Med. (2015) 372:1074–5. doi: 10.1056/NEJMc1500130, PMID: 25760364

[3] Cavanaugh C Okusa MD. The evolving role of novel biomarkers in glomerular disease: A review. Am J Kidney Dis. (2021) 77:122–31. doi: 10.1053/j.ajkd.2020.06.016, PMID: 33077315

[4] Kistler AD Salant DJ. Complement activation and effector pathways in membranous nephropathy. Kidney Int. (2024) 105:473–83. doi: 10.1016/j.kint.2023.10.035, PMID: 38142037

[5] Stangou MJ Marinaki S Papachristou E Liapis G Pateinakis P Gakiopoulou H. Histological grading in primary membranous nephropathy is essential for clinical management and predicts outcome of patients. Histopathology. (2019) 75:660–71. doi: 10.1111/his.13955, PMID: 31318463 PMC6856983

[6] Liu J Zha Y Zhang P He P He L. The association between serum complement 4 and kidney disease progression in idiopathic membranous nephropathy: A multicenter retrospective cohort study. Front Immunol. (2022) 13:896654. doi: 10.3389/fimmu.2022.896654, PMID: 35707542 PMC9189306

[7] Wu H Li X Li H. Gene fusions and chimeric RNAs, and their implications in cancer. Genes Dis. (2019) 6:385–90. doi: 10.1016/j.gendis.2019.08.002, PMID: 31832518 PMC6889028

[8] Li X Wang C-Y. From bulk, single-cell to spatial RNA sequencing. Int J Oral Sci. (2021) 13:1–6. doi: 10.1038/s41368-021-00146-0, PMID: 34782601 PMC8593179

[9] Mahbub SB Nguyen LT Habibalahi A Campbell JM Anwer AG Qadri UM. Non-invasive assessment of exfoliated kidney cells extracted from urine using multispectral autofluorescence features. Sci Rep. (2021) 11:10655. doi: 10.1038/s41598-021-89758-4, PMID: 34017033 PMC8138006

[10] Seegmiller JC Bachmann LM. Urine albumin measurements in clinical diagnostics. Clin Chem. (2024) 70:382–91. doi: 10.1093/clinchem/hvad174, PMID: 38321881

[11] Latt KZ Heymann J Jessee JH Rosenberg AZ Berthier CC Arazi A. Urine single-cell RNA sequencing in focal segmental glomerulosclerosis reveals inflammatory signatures. Kidney Int Rep. (2022) 7:289–304. doi: 10.1016/j.ekir.2021.11.005, PMID: 35155868 PMC8821042

[12] Fagerberg L Hallström BM Oksvold P Kampf C Djureinovic D Odeberg J. Analysis of the human tissue-specific expression by genome-wide integration of transcriptomics and antibody-based proteomics. Mol Cell Proteomics. (2014) 13:397–406. doi: 10.1074/mcp.M113.035600, PMID: 24309898 PMC3916642

[13] Xie Y Sakatsume M Nishi S Narita I Arakawa M Gejyo F. Expression, roles, receptors, and regulation of osteopontin in the kidney. Kidney Int. (2001) 60:1645–57. doi: 10.1046/j.1523-1755.2001.00032.x, PMID: 11703581

[14] Zhang Z Liu B Lin Z Mei L Chen R Li Z. SPP1 could be an immunological and prognostic biomarker: From pan-cancer comprehensive analysis to osteosarcoma validation. FASEB J. (2024) 38:e23783. doi: 10.1096/fj.202400622RR, PMID: 39037571

[15] De Schepper S Ge JZ Crowley G Ferreira LSS Garceau D Toomey CE. Perivascular cells induce microglial phagocytic states and synaptic engulfment via SPP1 in mouse models of Alzheimer’s disease. Nat Neurosci. (2023) 26:406–15. doi: 10.1038/s41593-023-01257-z, PMID: 36747024 PMC9991912

[16] Yang X Liu Z Zhou J Guo J Han T Liu Y. SPP1 promotes the polarization of M2 macrophages through the Jak2/Stat3 signaling pathway and accelerates the progression of idiopathic pulmonary fibrosis. Int J Mol Med. (2024) 54:89. doi: 10.3892/ijmm.2024.5413, PMID: 39129313 PMC11335352

[17] Ding H Xu Z Lu Y Yuan Q Li J Sun Q. Kidney fibrosis molecular mechanisms Spp1 influences fibroblast activity through transforming growth factor beta smad signaling. iScience. (2024) 27:109839. doi: 10.1016/j.isci.2024.109839, PMID: 39323737 PMC11422156

[18] Hoeft K Schaefer GJL Kim H Schumacher D Bleckwehl T Long Q. Platelet-instructed SPP1+ macrophages drive myofibroblast activation in fibrosis in a CXCL4-dependent manner. Cell Rep. (2023) 42:112131. doi: 10.1016/j.celrep.2023.112131, PMID: 36807143 PMC9992450

[19] Couser WG. Primary membranous nephropathy. Clin J Am Soc Nephrol. (2017) 12:983–97. doi: 10.2215/CJN.11761116, PMID: 28550082 PMC5460716

[20] Sethi S. New ‘Antigens’ in membranous nephropathy. J Am Soc Nephrol. (2021) 32:268–78. doi: 10.1681/ASN.2020071082, PMID: 33380523 PMC8054892

[21] Tao Q Liu N Wu J Chen J Chen X Peng C. Mefloquine enhances the efficacy of anti-PD-1 immunotherapy via IFN-γ-STAT1-IRF1-LPCAT3-induced ferroptosis in tumors. J Immunother Cancer. (2024) 12:e008554. doi: 10.1136/jitc-2023-008554, PMID: 38471712 PMC10936479

[22] Rood IM Merchant ML Wilkey DW Zhang T Zabrouskov V van der Vlag J. Increased expression of lysosome membrane protein 2 in glomeruli of patients with idiopathic membranous nephropathy. Proteomics. (2015) 15:3722–30. doi: 10.1002/pmic.201500127, PMID: 26304790

[23] Ronco P Plaisier E. Time to abandon kidney biopsy to diagnose membranous nephropathy? CJASN. (2021) 16:1787–9. doi: 10.2215/CJN.11180821, PMID: 34782362 PMC8729492

[24] Cavanaugh C Perazella MA. Urine sediment examination in the diagnosis and management of kidney disease: core curriculum 2019. Am J Kidney Dis. (2019) 73:258–72. doi: 10.1053/j.ajkd.2018.07.012, PMID: 30249419

[25] Inoue CN Sunagawa N Morimoto T Ohnuma S Katsushima F Nishio T. Reconstruction of tubular structures in three-dimensional collagen gel culture using proximal tubular epithelial cells voided in human urine. In Vitro Cell Dev Biol Anim. (2003) 39:364–7. doi: 10.1290/1543-706X(2003)039<0364:ROTSIT>2.0.CO;2, PMID: 15038777

[26] Al-Malki AL. Assessment of urinary osteopontin in association with podocyte for early predication of nephropathy in diabetic patients. Dis Markers. (2014) 2014:493736. doi: 10.1155/2014/493736, PMID: 24876663 PMC4024407

[27] Bharadwaj S Liu G Shi Y Wu R Yang B He T. Multipotential differentiation of human urine-derived stem cells: Potential for therapeutic applications in urology. Stem Cells. (2013) 31:1840–56. doi: 10.1002/stem.1424, PMID: 23666768

[28] Rm C. Biomarker definitions and their applications. Exp Biol Med (Maywood NJ). (2018) 243 (3):213–21. doi: 10.1177/1535370217750088, PMID: 29405771 PMC5813875

[29] Ix JH Shlipak MG. The promise of tubule biomarkers in kidney disease: A review. Am J Kidney Dis. (2021) 78:719–27. doi: 10.1053/j.ajkd.2021.03.026, PMID: 34051308 PMC8545710

[30] Zhou S Ai Z Li W You P Wu C Li L. Deciphering the pharmacological mechanisms of taohe-chengqi decoction extract against renal fibrosis through integrating network pharmacology and experimental validation *in vitro* and *in vivo* . Front Pharmacol. (2020) 11:425. doi: 10.3389/fphar.2020.00425, PMID: 32372953 PMC7176980

[31] Guo W Huang J Wang N Tan H-Y Cheung F Chen F. Integrating network pharmacology and pharmacological evaluation for deciphering the action mechanism of herbal formula zuojin pill in suppressing hepatocellular carcinoma. Front Pharmacol. (2019) 10:1185. doi: 10.3389/fphar.2019.01185, PMID: 31649545 PMC6795061

[32] Keller SA Chen Z Gaponova A Korzinkin M Berquez M Luciani A. Drug discovery and therapeutic perspectives for proximal tubulopathies. Kidney Int. (2023) 104:1103–12. doi: 10.1016/j.kint.2023.08.026, PMID: 37783447

[33] Han R Hu S Qin W Shi J Hou Q Wang X. C3a and suPAR drive versican V1 expression in tubular cells of focal segmental glomerulosclerosis. JCI Insight. (2019) 4:e122912. doi: 10.1172/jci.insight.122912, PMID: 31292294 PMC6629242

[34] Gould SE Day M Jones SS Dorai H. BMP-7 regulates chemokine, cytokine, and hemodynamic gene expression in proximal tubule cells. Kidney Int. (2002) 61:51–60. doi: 10.1046/j.1523-1755.2002.00103.x, PMID: 11786084

[35] Morigi M Macconi D Zoja C Donadelli R Buelli S Zanchi C. Protein overload-induced NF-kappaB activation in proximal tubular cells requires H(2)O(2) through a PKC-dependent pathway. J Am Soc Nephrol. (2002) 13:1179–89. doi: 10.1097/01.ASN.0000013304.48222.02, PMID: 11961005

[36] Gewin LS. Renal fibrosis: primacy of the proximal tubule. Matrix Biol. (2018) 68–69:248–62. doi: 10.1016/j.matbio.2018.02.006, PMID: 29425694 PMC6015527

[37] Brennan EP Cacace A Godson C. Specialized pro-resolving mediators in renal fibrosis. Mol Aspects Med. (2017) 58:102–13. doi: 10.1016/j.mam.2017.05.001, PMID: 28479307

[38] Meng X-M. Inflammatory mediators and renal fibrosis. Adv Exp Med Biol. (2019) 1165:381–406. doi: 10.1007/978-981-13-8871-2_18, PMID: 31399975

[39] Foresto-Neto O Menezes-Silva L Leite JA Andrade-Silva M Câmara NOS. Immunology of kidney disease. Annu Rev Immunol. (2024) 42:207–33. doi: 10.1146/annurev-immunol-090122-045843, PMID: 38211945

[40] Yang S Plotnikov SV. Mechanosensitive regulation of fibrosis. Cells. (2021) 10:994. doi: 10.3390/cells10050994, PMID: 33922651 PMC8145148

[41] Ronco P Beck L Debiec H Fervenza FC Hou FF Jha V. Membranous nephropathy. Nat Rev Dis Primers. (2021) 7:69. doi: 10.1038/s41572-021-00303-z, PMID: 34593809

[42] Liu B-C Tang T-T Lv L-L Lan H-Y. Renal tubule injury: a driving force toward chronic kidney disease. Kidney Int. (2018) 93:568–79. doi: 10.1016/j.kint.2017.09.033, PMID: 29361307

[43] Chevalier RL. The proximal tubule is the primary target of injury and progression of kidney disease: role of the glomerulotubular junction. Am J Physiol Renal Physiol. (2016) 311:F145–161. doi: 10.1152/ajprenal.00164.2016, PMID: 27194714 PMC4967168

[44] Wei J Marisetty A Schrand B Gabrusiewicz K Hashimoto Y Ott M. Osteopontin mediates glioblastoma-associated macrophage infiltration and is a potential therapeutic target. J Clin Invest. (2019) 129:137–49. doi: 10.1172/JCI121266, PMID: 30307407 PMC6307970

[45] Kumari A Kashyap D Garg VK. Chapter Three - Osteopontin in cancer. In: Makowski GS, editor. Advances in Clinical Chemistry. Amsterdam: Elsevier (2024). p. 87–110. doi: 10.1016/bs.acc.2023.11.002, PMID: 38280808

[46] Bill R Wirapati P Messemaker M Roh W Zitti B Duval F. CXCL9:SPP1 macrophage polarity identifies a network of cellular programs that control human cancers. Science. (2023) 381:515–24. doi: 10.1126/science.ade2292, PMID: 37535729 PMC10755760

[47] Li H Li P Shen Q Zhu Z Yang M Zhang X. Nfil3 contributes to renal fibrosis by activating fibroblasts through directly promoting the expression of Spp1. Biochim Biophys Acta Mol Basis Dis. (2025) 1871:167741. doi: 10.1016/j.bbadis.2025.167741, PMID: 39986442

