# Supplementary figures and images for: SPP1 as a biomarker for idiopathic membranous nephropathy progression and its regulatory role in inflammation and fibrosis
Source: Front Immunol. 2025 Sep 26;16:1671891. doi: 10.3389/fimmu.2025.1671891 (PMC12510867; doi:10.3389/fimmu.2025.1671891)

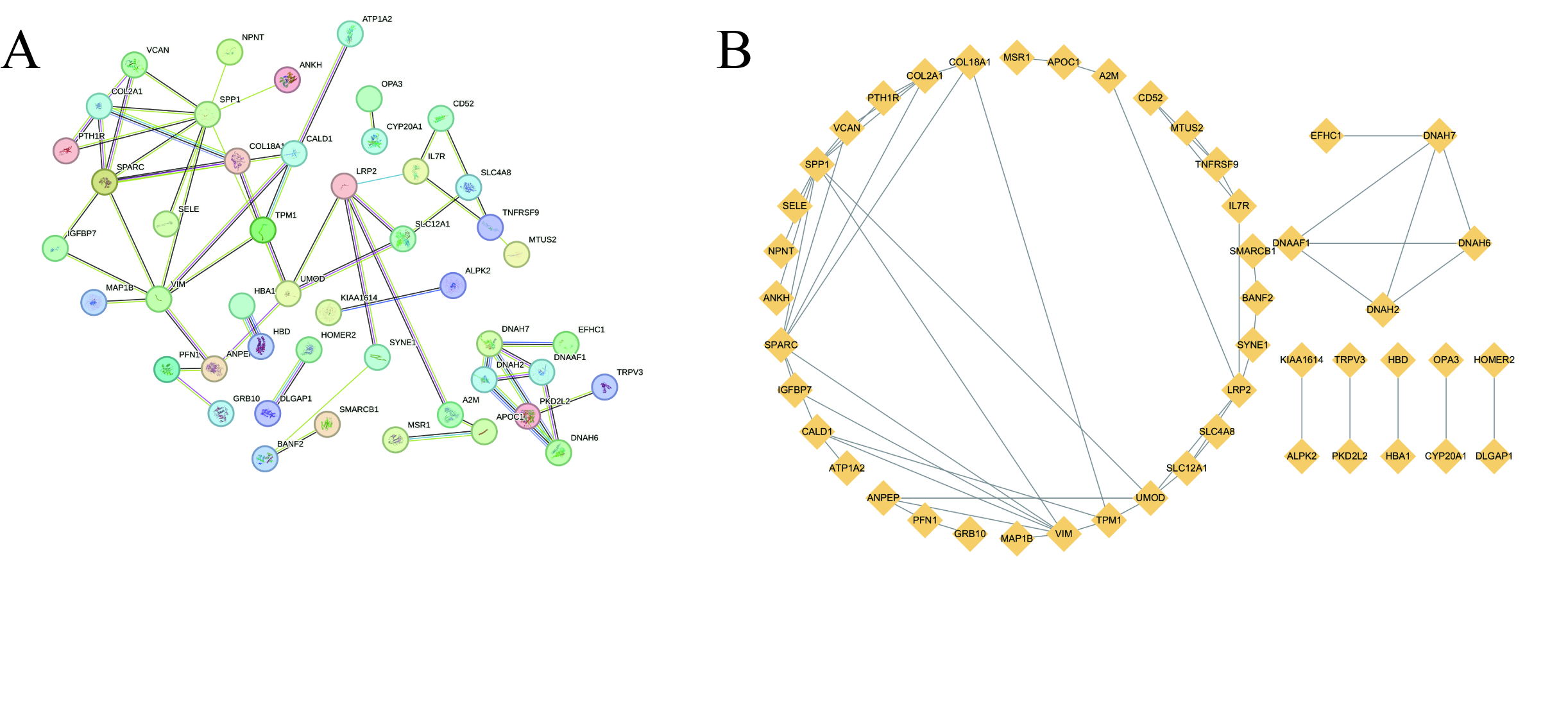

Supplement: Supplementary Figure 1 — PPI network of co-upregulated genes across three urine samples. [file Image1.tif]

**nFeature\_RNA**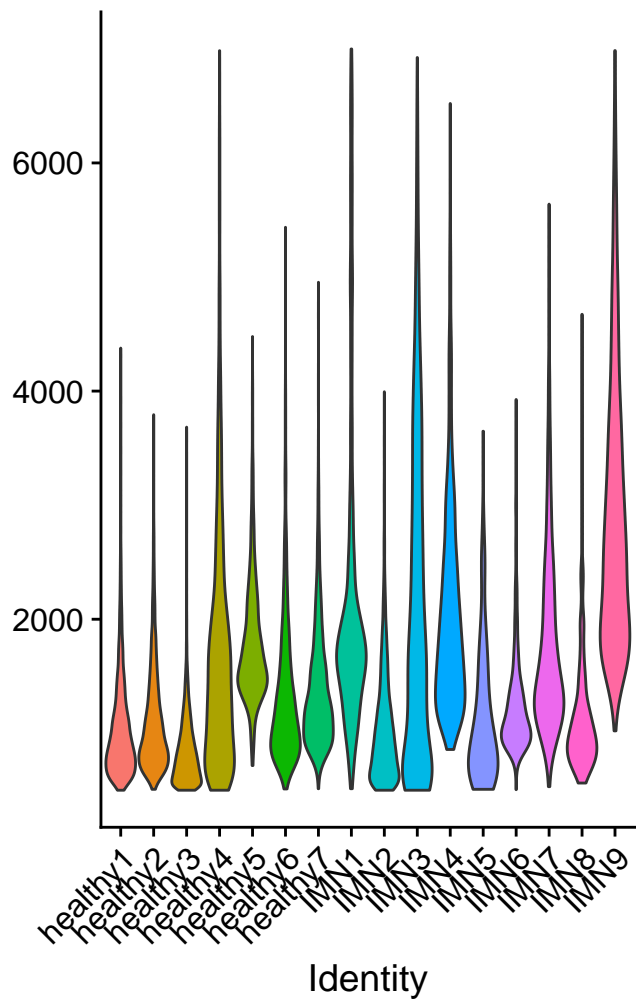**nCount\_RNA**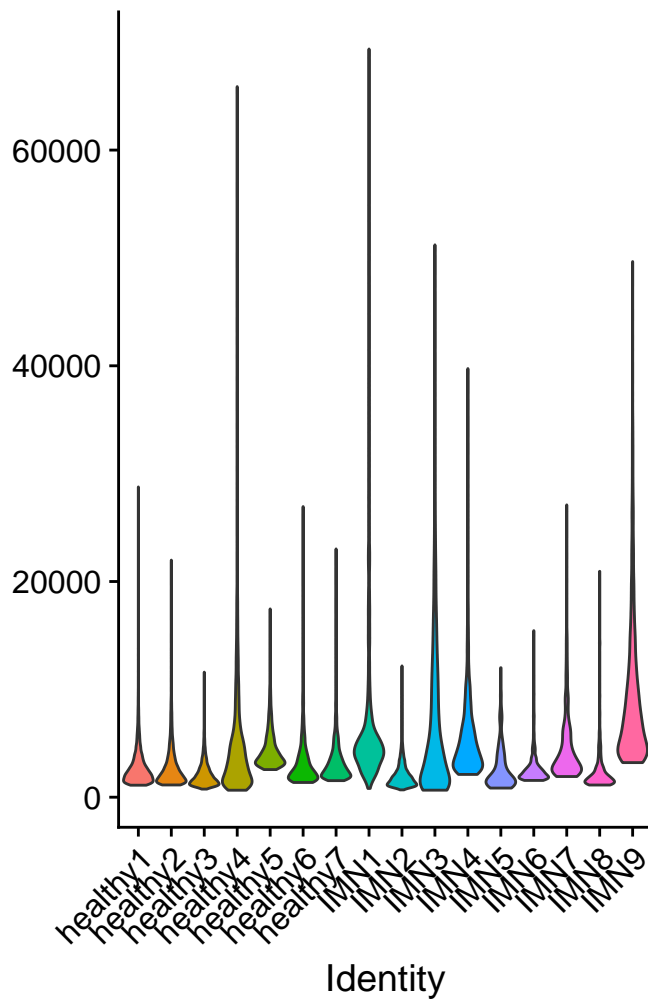**percent.mt**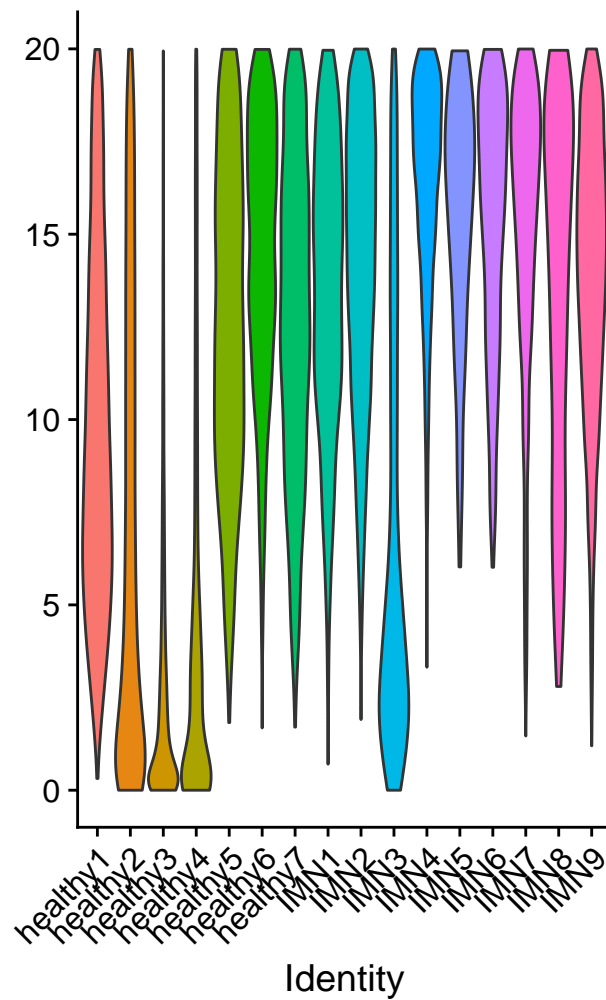

Supplement: Supplementary Figure 2 — scRNA-seq quality control plots of 9 IMN renal tissues and 7 normal renal tissues. [file Image2.pdf]
