# Supplementary material for: SPP1 as a biomarker for idiopathic membranous nephropathy progression and its regulatory role in inflammation and fibrosis
Source: Front Immunol. 2025 Sep 26;16:1671891. doi: 10.3389/fimmu.2025.1671891 (PMC12510867; doi:10.3389/fimmu.2025.1671891)
Supplement: Supplementary Table 2 — Table of primer sequences used in this study. [file Table2.docx]

Primer sequences

| Gene name | Forward primer (5' to 3') | Reverse primer (5' to 3') |
| --- | --- | --- |
| β-actin | TCACCATGGATGATGATATCGC | CCACATAGGAATCCTTCTGACC |
| SPP1 | ATCACCTGTGCCATACCAGT | GAGATGGGTCAGGGTTTAGCC |
| TNF-α | CCTGCTGCACTTTGGAGTGAT | CAGCTTGAGGGTTTGCTACAAC |
| IL-1β | TGATGGCTTATTACAGTGGCA | TGGTGGTCGGAGATTCGT |
| TGF-β1 | AACCCACAACGAAATCTATG | CCACTTTTAACTTGAGCCTC |
| Fibronectin | CTCCCAGAGAAGTGGTCCCT | GAGAGCTTCTTGTCCTGTCTT |
| Collagen I | AAGGTGTTGTGCGATGACG | TTGGTCGGTGGGTGACTCT |
| Vimentin | TGAACGCAAAGTGGAATC | GGTCAGGCTTGGAAACAT |
| NR2F1 | AAAGCCATCGTGCTGTTCA | TGGGGGTTTTACCTACCAAA |
