# Supplementary material for: SPP1 as a biomarker for idiopathic membranous nephropathy progression and its regulatory role in inflammation and fibrosis
Source: Front Immunol. 2025 Sep 26;16:1671891. doi: 10.3389/fimmu.2025.1671891 (PMC12510867; doi:10.3389/fimmu.2025.1671891)
Supplement: Supplementary Table 3 — Detailed table of antibodies used in Western Blot. [file Table3.docx]

| Primary Antibody | Brand of Primary Antibody | Working Concentration of Primary Antibody | Primary Antibody Incubation Parameters |
| --- | --- | --- | --- |
| beta III tubulin | Servicebio, Wuhan, China, GB12139-50 | 1:10000 | overnight at 4°C |
| β-actin | Abways, Shanghai, China, AB0035 | 1:10000 | overnight at 4°C |
| NR2F1 | Abways, Shanghai, China, CY7048 | 1:1000 | overnight at 4°C |
| Osteopontin | Abcam, Cambridge, UK, ab283656 | 1:1000 | overnight at 4°C |
| Fibronectin | Abcam, Cambridge, UK, ab268020 | 1:1000 | overnight at 4°C |
| TNF alpha | Abcam, Cambridge, UK, ab307164 | 1:1000 | overnight at 4°C |

Detailed Table of Antibodies Used in Western Blot

| Secondary Antibody | Brand of Secondary Antibody | Working Concentration of Secondary Antibody | Secondary Antibody Incubation Parameters |
| --- | --- | --- | --- |
| Sheep Anti-Mouse IgG-HRP | Cell Signaling Technology, Massachusetts, USA,7076P2 | 1:1000 | 1 hour at room temperature |
| Sheep Anti- Rabbit IgG-HRP | Cell Signaling Technology, Massachusetts, USA,7074P2 | 1:1000 | 1 hour at room temperature |
