# Supplementary material for: SPP1 as a biomarker for idiopathic membranous nephropathy progression and its regulatory role in inflammation and fibrosis
Source: Front Immunol. 2025 Sep 26;16:1671891. doi: 10.3389/fimmu.2025.1671891 (PMC12510867; doi:10.3389/fimmu.2025.1671891)
Supplement: Supplementary Table 4 — Detailed table of antibodies used in Immunohistochemistry. [file Table4.docx]

| Primary Antibody | Brand of Primary Antibody | Working Concentration of Primary Antibody | Primary Antibody Incubation Parameters |
| --- | --- | --- | --- |
| Osteopontin | Abcam, Cambridge, UK, ab283656 | 1:2000 | overnight at 4°C |
| Fibronectin | Abcam, Cambridge, UK, ab268020 | 1:2000 | overnight at 4°C |
| TNF alpha | ZENBIO, Sichuan, China, 346654 | 1:50 | overnight at 4°C |

Detailed Table of Antibodies Used in Immunohistochemistry

| Secondary Antibody | Brand of Secondary Antibody | Working Concentration of Secondary Antibody | Secondary Antibody Incubation Parameters |
| --- | --- | --- | --- |
| Goat Anti-Rabbit/Mouse HRP-Labeled Polymer | Proteintech, Hubei, China, PK10006 | Ready-to-Use Antibody | 1 hour at room temperature |
