# Supplementary material for: SPP1 as a biomarker for idiopathic membranous nephropathy progression and its regulatory role in inflammation and fibrosis
Source: Front Immunol. 2025 Sep 26;16:1671891. doi: 10.3389/fimmu.2025.1671891 (PMC12510867; doi:10.3389/fimmu.2025.1671891)
Supplement: Supplementary Table 5 — Detailed table of antibodies used in Immunofluorescence. [file Table5.docx]

| Primary Antibody | Brand of Primary Antibody | Working Concentration of Primary Antibody | Primary Antibody Incubation Parameters |
| --- | --- | --- | --- |
| Osteopontin | Servicebio, Wuhan, China, GB122328-50 | 1:700 | overnight at 4°C |
| Fibronectin | Abcam, Cambridge, UK, ab268020 | 1:50 | overnight at 4°C |
| TNF alpha | ZENBIO, Sichuan, China, 346654 | 1:50 | overnight at 4°C |
| NR2F1 | Abways, Shanghai, China, CY7048 | 1:50 | overnight at 4°C |

Detailed Table of Antibodies Used in Immunofluorescence

| Secondary Antibody | Brand of Secondary Antibody | Working Concentration of Secondary Antibody | Secondary Antibody Incubation Parameters |
| --- | --- | --- | --- |
| Cy3-Conjugated Goat Anti-Rat IgG | Servicebio, Wuhan, China, GB21302 | 1:200 | 1 hour at room temperature |
| Alexa Fluor 488-Conjugated Goat Anti-Rabbit IgG | Servicebio, Wuhan, China, GB25303 | 1:200 | 1 hour at room temperature |
